# Advanced Nanotechnologies for Extracellular Vesicle‐Based Liquid Biopsy

**DOI:** 10.1002/advs.202102789

**Published:** 2021-08-31

**Authors:** Li Min, Binshuai Wang, Han Bao, Xinran Li, Libo Zhao, Jingxin Meng, Shutao Wang

**Affiliations:** ^1^ Department of Gastroenterology Beijing Friendship Hospital Capital Medical University National Clinical Research Center for Digestive Diseases Beijing Digestive Disease Center Beijing Key Laboratory for Precancerous Lesion of Digestive Disease Beijing 100050 P. R. China; ^2^ Department of Urology Peking University Third Hospital Beijing 100191 P. R. China; ^3^ Key Laboratory of Bio‐inspired Materials and Interfacial Science CAS Center for Excellence in Nanoscience Technical Institute of Physics and Chemistry Chinese Academy of Sciences Beijing 100190 P. R. China; ^4^ University of Chinese Academy of Sciences Beijing 100049 P. R. China; ^5^ Echo Biotech Co., Ltd. Beijing 102206 P. R. China

**Keywords:** cancer, disease diagnosis, extracellular vesicles, liquid biopsy, nanotechnologies

## Abstract

Extracellular vesicles (EVs) are emerging as a new source of biomarkers in liquid biopsy because of their wide presence in most body fluids and their ability to load cargoes from disease‐related cells. Owing to the crucial role of EVs in disease diagnosis and treatment, significant efforts have been made to isolate, detect, and analyze EVs with high efficiency. A recent overview of advanced EV detection nanotechnologies is discussed here. First, several key challenges in EV‐based liquid biopsies are introduced. Then, the related pivotal advances in nanotechnologies for EV isolation based on physical features, chemical affinity, and the combination of nanostructures and chemical affinity are summarized. Next, a summary of high‐sensitivity sensors for EV detection and advanced approaches for single EV detection are provided. Later, EV analysis is introduced in practical clinical scenarios, and the application of machine learning in this field is highlighted. Finally, future opportunities for the development of next‐generation nanotechnologies for EV detection are presented.

## Graphical abstract

Extracellular vesicles (EVs) have attracted interest as an emerging source of disease biomarkers. Advanced nanotechnologies are presented here to detect EVs in body fluids as well as their potential clinical applications to build a bridge between material scientists and clinicians. Moreover, future opportunities are also pointed out to develop next‐generation nanotechnologies for EV detection.

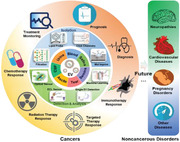

## 1 Introduction

Liquid biopsy, which refers to the sampling of nonsolid tissues, could be performed more frequently due to minimal invasion compared to traditional tissue biopsy.^[^ ^1^ ^]^ There are three main types of analytes for liquid biopsy: circulating tumor cells (CTCs), cell‐free DNA (cfDNA), and extracellular vesicles (EVs). CTCs are tumor cells that shed from primary or metastatic tumor sites, rendering them promising for cancer diagnosis and monitoring. However, the rare number of CTCs in the blood causes huge difficulties in their enrichment.^[^ ^2^, ^3^ ^]^ cfDNA refers to the free DNA fragments released into the blood. The detection of cfDNA is commercially available for cancer treatment monitoring and noninvasive prenatal testing.^[^ ^4^ ^]^ However, a large amount of nonspecific cfDNA co‐isolated with disease‐specific cfDNA would interfere with downstream assays.^[^ ^5^ ^]^ Compared with CTCs and cfDNA, EVs exhibit advantages such as high abundance and high stability.^[^ ^6^ ^]^ EVs derived from different cancer cells were found to participate in the formation of the premetastatic niche,^[^ ^7^ ^]^ inhibit the response to immunotherapy,^[^ ^8^ ^]^ and promote tumor angiogenesis.^[^ ^9^ ^]^ The presence of disease‐specific EVs in the microenvironment renders them ideal targets for liquid biopsy. Additionally, bioactive cargoes encapsulated in EVs, such as proteins, RNAs, and DNAs, contain largely enriched information, thereby enabling their potential as disease biomarkers.^[^ ^10^ ^]^

Many efforts have been made to achieve sensitive, accurate, and cost‐effective detection of EVs in human body fluids. Several excellent reviews have focused on the advances in EV‐associated biological processes^[^ ^11^, ^12^ ^]^ and EV‐based technologies^[^ ^13^, ^14^ ^]^ from the individual viewpoint of clinicians and materials scientists, respectively. An interdisciplinary overview of EV‐based liquid biopsy is needed to bridge advanced nanotechnologies and clinical scenarios. Herein, we introduce the key advances in recent nanotechnologies for EV‐based liquid biopsy. First, we present several important challenges to detect body fluid‐derived EVs. Second, we summarize various advanced nanotechnologies in EV isolation. Third, we introduce the development of high‐sensitivity sensors and single EV detection approaches. Fourth, we present the possible clinical scenarios to build a bridge between materials scientists and clinicians and highlight the application of machine learning in EV analysis. Finally, we present a future perspective on the development of next‐generation technologies for EV detection.

## 2 Knowledge of EVs in Body Fluids

The origin of the EV study can be traced to Charles Darwin's theory of Pangenesis, which was published in 1868. Darwin proposed small particles called “gemmules” diffusing into recipient cells, which mediated natural selection‐induced variations.^[^ ^15^ ^]^ The existence of extracellular particles carrying information remains uncertain for nearly a century until Chargaff et al. reported the first effect of these hypothetical particles by discovering the procoagulant function of the sediment of plasma by ultracentrifugation (UC).^[^ ^16^ ^]^ EVs were first observed by Peter Wolf by electron microscopy in 1967,^[^ ^17^ ^]^ and the primary theory of the endosomal origin of EVs was proposed in 1983 by Pan^[^ ^18^ ^]^ and Harding.^[^ ^19^ ^]^ Moreover, the biological functions of EVs (e.g., antigen presentation) were first identified in B lymphocytes in 1996.^[^ ^20^ ^]^ Various functions of EVs in cell­to­cell communication have been widely reported since the beginning of the 21st century,^[^ ^7^, ^21^, ^22^ ^]^ setting off an upsurge in EV study.

EVs can mainly be divided into three categories based on origin and size distribution: endosomal‐originated exosomes (50–150 nm), plasma membrane budding microvesicles (100–1000 nm), and apoptosis‐induced apoptotic bodies (100–5000 nm).^[^ ^12^ ^]^ To indicate the specific origin or characteristics, other names (e.g., prostasomes, oncosomes, and dexosomes) have also been used by researchers. Regardless of the origin and other features, EVs have been employed as the only nomenclature generic term in this review.

### 2.1 Characterization of EVs in Human Body Fluids

**Figure 1:**
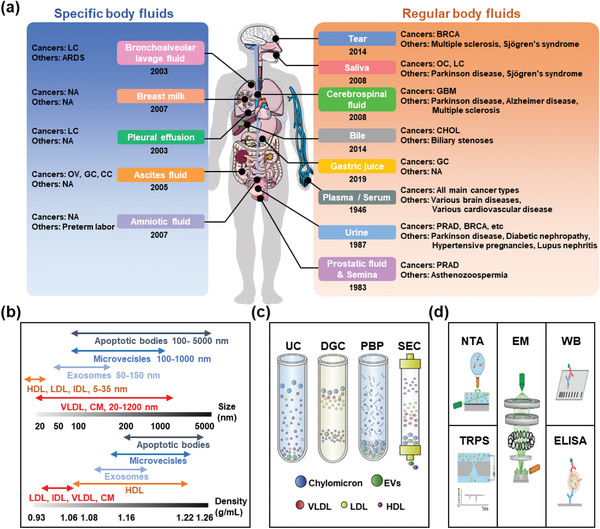
Knowledge of EVs in human body fluids. a) Milestones of EV isolation and detection from human body fluids including specific body fluids (e.g., bronchoalveolar lavage fluid) and regular body fluids (e.g., tear). Through detecting EVs from different body fluids, various diseases (e.g., cancer) can be diagnosed. b) Compared to other blood‐derived nanoparticles, EVs show different distributions of size and density. Reproduced with permission.^[^ ^12^ ^]^ Copyright 2019, Springer Nature. c) Traditional approaches for EV isolation: UC, DGC, PBP, and SEC. Reproduced with permission.^[^ ^35^ ^]^ Copyright 2019, Elsevier. d) Traditional approaches for EV detection: NTA, TRPS, EM, WB, and ELISA. Reproduced with permission.^[^ ^50^ ^]^ Copyright 2018, Wiley‐VCH. BRCA: breast cancer; GBM: glioblastoma multiform; GC: gastric cancer; CC: colon cancer; LC: lung cancer; OV: ovarian cancer; PRAD: prostatic adenocarcinoma; OC: oral carcinoma; CHOL: cholangiocarcinoma; ARDS: acute respiratory distress syndrome. HDL: high‐density lipoprotein; LDL: low‐density lipoprotein; IDL: intermediate‐density lipoprotein; VLDL: very low‐density lipoprotein; CM: chylomicrons; UC: ultracentrifugation; DGC: density gradient centrifugation; PBP: polymer‐based precipitation; SEC: size exclusion chromatography; NTA: nanoparticle tracking analysis; TRPS: tunable resistive pulse sensing; EM: electron microscopy; WB: western blotting; ELISA: enzyme‐linked immunosorbent assay.

EVs were first identified in human plasma in 1946.^[^ ^16^ ^]^ Thereafter, Wiggins et al.^[^ ^23^ ^]^ and Brody et al.^[^ ^24^ ^]^ characterized EVs in prostatic fluid and urine in the 1980s. Subsequently, EVs were detected in most human body fluids, including lavage fluid,^[^ ^25^ ^]^ pleural effusion,^[^ ^26^ ^]^ ascites fluid,^[^ ^27^ ^]^ amniotic fluid,^[^ ^28^ ^]^ breast milk,^[^ ^29^ ^]^ saliva,^[^ ^30^ ^]^ cerebrospinal fluid,^[^ ^31^ ^]^ bile,^[^ ^32^ ^]^ tears,^[^ ^33^ ^]^ and gastric juice.^[^ ^34^ ^]^ These body fluids can be roughly divided into two categories: specific body fluids and regular body fluids. Specific body fluids (e.g., bronchoalveolar lavage fluid) are present only under specific pathological or physiological conditions. The detection of related EVs is always focused on few diseases, such as lung cancer and acute respiratory distress syndrome (**Figure** **1**a, left). On the other hand, regular body fluids (e.g., tears) are present at any time. They reflect the health or disease status of their original locations, referring to many disorders such as breast cancer and Parkinson's disease (Figure 1a, right). In particular, blood (i.e., plasma/serum) is a very promising source of biomarkers that provides efficient and sufficient information on the general health status. Therefore, EVs from various human body fluids can be considered as promising biomarkers of different diseases.

EV concentrations in body fluids can be commonly regarded as the basic parameter in EV‐based liquid biopsies. However, the determination of EV concentrations is largely affected by isolation technology and quantification methods. According to previous reports,^[^ ^35^ ^]^ human plasma conceived a general concentration of EVs ranging from 10^7^ to 10^13^ particles mL^−1^. When isolating EVs by UC and quantifying them by nanoparticle tracking analysis (NTA), the range of plasma EV concentration can be shrunk to 10^9^–10^11^ particles mL^−1^.^[^ ^35^ ^]^ Considering 10^13^–10^16^ particles mL^−1^ lipoprotein and chylomicron in plasma, the isolation approaches of EVs based on their physical and chemical properties should have a 1000‐fold higher efficiency than those dopants to obtain an acceptable purity in EV‐enriched fractions (Figure 1b).^[^ ^36^ ^]^ Compared with plasma, EV concentrations from other body fluids are even more unreliable.

In addition to the concentration, the characteristics of EVs, such as shape, density, and zeta potential, should also be considered. A bowl‐shaped morphology was observed, which was caused by dehydration during the preparation of EV‐enriched samples. There is no consensus on the density and zeta potential of EVs owing to the strong heterogeneity in EV subpopulations. With a density of 1.12–1.21 g mL^−1^, EVs showed a vague and overlapping density among different subgroups. For instance, Théry's team divided EVs into four subgroups based on the size (100 K pellet vs 10 K pellet) and density (1.115 g mL^−1^ F3 fraction vs 1.145 g mL^−1^ F5 fraction) and reported a distinct protein profile of each EV subgroup.^[^ ^37^ ^]^ For the reason that density gradient centrifugation (DGC) is commonly used for EV isolation, the heterogeneity of densities should also be considered (Figure 1b). In addition, the negative zeta potential of EVs (i.e., −15 ± 2 mV) has been reported in most studies. The fine separation of different EV subsets provided evidence of the heterogeneity in zeta potential,^[^ ^38^ ^]^ indicating an additional consideration of the electrophoresis‐based separation of EVs.

Plasma always exhibits the advantages of universal availability and high stability. In contrast, the heterogeneous physical and chemical characteristics of other body fluids may cause problems in EV detection. For example, extreme acidic conditions of gastric juice (pH = 2.0) largely threaten the EV integrity in traditional EV isolation procedures, and the high viscosity of bile makes it difficult to isolate EVs directly by filtration or centrifugation. Additionally, the concentration of urine and saliva is easily affected by different physiological conditions, and the timing of sample collection largely affects the results of EV detection. Understanding the physical and chemical characteristics of different body fluids and the development of adaptive nanotechnologies would provide more opportunities for EV‐based liquid biopsy.

In summary, EVs have been identified in most human body fluids, but the definite concentrations of EVs are still controversial. Moreover, considering the heterogeneity of different EV subsets in different body fluids, there are still various challenges in EV detection.

### 2.2 Traditional Approaches for EV Isolation

There are insurmountable barriers to the accurate detection of body fluid‐derived EVs. The most widely known barrier is the low concentration of EVs in circulation. Therefore, we usually need to isolate EVs from 1000‐fold abundant impurities with similar properties (e.g., size and density) before analysis.^[^ ^35^ ^]^ Traditional EV isolation technologies are mainly based on the physical properties of EVs, such as size, density, and solubility. In the following sections, we have introduced the four most widely used EV isolation technologies (Figure 1c): UC, DGC, polymer‐based precipitation (PBP), and size exclusion chromatography (SEC).

UC is the most commonly used EV isolation approach. According to the difference in sedimentation coefficients, particles of different sizes and densities are separated in the liquid. Thus, EVs are precipitated at a high speed (≈100 000 × *g*), while large cell debris and other unwanted particles are removed at a low speed. Many factors affect the amount and purity of the EV‐enriched fraction isolated by UC. For example, more dilution‐centrifugation cycles would result in a purer EV outcome and lower recovery. Thus, a trade‐off between purity and yield should be considered according to downstream analysis.^[^ ^39^ ^]^ The choice of different rotors should also be considered case by case. A larger rotor usually indicates a higher sample processing capacity and a lower maximum speed. Swinging‐bucket rotors are much more time‐consuming than fixed‐angle rotors, while providing better separation of individual particles tied from a mixture.^[^ ^40^ ^]^ To reduce the viscosity of the liquid before UC, the plasma should be diluted 1:7 with PBS when dealing with a small start‐up sample volume.^[^ ^10^ ^]^ Although it is commonly believed that professional skills are rarely needed, the UC isolation protocol still needs to be optimized according to the input volumes, type of samples, and rotor types.

DGC is a refined centrifugation‐based procedure for EV isolation. When every particle moves to a balance between centrifugal force and buoyancy in a density gradient solution, the EVs can be isolated from other particles owing to their different densities. Sucrose solutions with density gradients are widely used in DGC,^[^ ^41^ ^]^ and an improved DGC pipeline with iodixanol was proposed to reduce the damage to the biological activity of EVs.^[^ ^42^ ^]^ Additionally, only swinging‐bucket rotors are suitable for DGC to maintain a gradient parallel to the direction of the imposed force. Generally, DGC can yield a higher purity than UC but requires substantial time, biofluid volume, and expertise, largely limiting point‐of‐care clinical translation.

PBP was a popular approach that offered an “off the shelf” EV isolation with low input volumes. Adding highly hydrophilic water‐excluding polymers dramatically decreases the solubility of EVs and facilitates the precipitation of EVs under low‐speed centrifugation. Polyethylene glycol (PEG) is the most widely used reagents for EV precipitation, and a modified PEG/dextran aqueous two‐phase system was used to achieve a higher recovery.^[^ ^43^ ^]^ Many kits (e.g., ExoQuick, Total Exosome Isolation, and ExoSpin) have been invented and extensively promoted the outburst of EV studies because neither additional expertise nor expensive equipment was needed. Nonetheless, PBP methods have been severely criticized for troublesome contamination, as the polymers may also coprecipitate protein aggregates.

SEC can separate particles of different sizes based on the molecular sieve effect. For example, Boing et al. modified the SEC procedure and performed one‐step isolation of EVs from plasma.^[^ ^44^ ^]^ The improved SEC is easy to perform and is especially suitable for the removal of high‐density lipoprotein (HDL) in plasma. However, the purity of EVs isolated by one‐step SEC was still no match for EVs isolated by conventional UC and DGC. Many SEC‐based commercial kits (e.g., qEV Columns and Exosupur) are already available, but an improved design of packaged matrix ingredients is urgently needed.

In brief, there is no package solution for EV isolation. To improve the efficiency and quality of EV isolation, it is worthwhile to combine multiple methods. For instance, a homogeneous EV subpopulation could be obtained by combining UC with DGC, further dividing EV‐enriched fractions into subgroups according to their size and density.^[^ ^37^ ^]^ Moreover, compared to the single SEC, combining SEC with UC/DGC exhibited an improvement in purity.^[^ ^45^, ^46^ ^]^

### 2.3 Traditional Approaches for EV Detection

Generally, there are three categories of traditional EV detection approaches: quantification, visualization, and biochemical measurement. Traditional quantification methods include NTA, dynamic light scattering (DLS), and tunable resistive pulse sensing (TRPS), while traditional visualization methods include scanning electron microscopy (SEM), transmission electron microscopy (TEM), and cryo‐electron microscopy (cryo‐EM). According to the aim of the detection, biochemical measurement assays are largely customized including western blotting (WB), polymerase chain reaction (PCR), and enzyme­linked immunosorbent assay (ELISA). Here, we have provided a brief summary of these detection approaches and highlighted their defects and challenges (Figure 1d).

The NTA is a popular EV quantification technology, which directly records the trajectories of individual scattering EVs under a microscope and transforms their displacements to the sizes of EVs based on the Strokes–Einstein equation. However, the displacements of the EVs are estimated based on an approximation by projecting the 3D trajectories onto a 2D plane. Thus, the sizes of the EVs detected by NTA are inevitably distorted. Similarly, DLS provides the size distribution of EVs by analyzing the fluctuations in the intensity of scattered light produced by EVs upon illumination. Therefore, DLS provides a faster assessment of the size distribution, but not the concentration of EVs. TRPS detects the electrical signals generated by the change in ion conductance when an EV passes through a stretchable pore and transforms these signals into the size and concentration of EVs. Additionally, the performance of these traditional EV quantification technologies is largely dependent on the efficiency and purity of the aforementioned isolation approaches, which further restricts comparability and repeatability.^[^ ^35^ ^]^

Generally, EV visualization approaches are based on electron microscopy, such as SEM, TEM, and cryo‐EM. For SEM, the sample is scanned by an electron beam, and the secondary electrons ejected from the samples are collected, and the surface morphology is generated. For TEM, a focused electron beam is allowed to penetrate into the sample, and the image is created based on the interactions between the transmission electrons and the sample. The main problem of EV visualization by SEM and TEM is artificial perturbations resulting from sample handling procedures. For example, the frequently reported saucer‐shaped morphology of EVs is due to dehydration during sample processing. Nevertheless, cryo­EM imaging without fixation and dehydration indicates the actual spherical shape of EVs. Other artificial perturbations should also be noted in EV visualization. For instance, centrifugation with a high *g*‐force would lead to EV fusion, while adding chemical reagents could result in the loss of biological activity, shape change, and even rupture of EVs.^[^ ^11^, ^47^ ^]^

Biochemical measurement assays of EVs are highly customized according to the aim of detection, and the main barrier in these assays is the heterogeneity of EVs. Disease‐specific EV signals are masked by nonspecific EV signals, which largely restrict the effectiveness of regular en bloc EV detection. For example, a large number of EVs from platelets and megakaryocytes greatly interfere with the detection of EV‐derived biomarkers.^[^ ^48^ ^]^ Targeted capture of EVs from specific origins has been widely used in biomarker studies with specific antibodies,^[^ ^49^ ^]^ but a much lower gain of EVs would also restrict downstream detection. Apart from technical barriers, how clinical factors (e.g., age and sex) affect the quantity and content of circulating EVs is largely unknown. Thus, these factors are not only confounders in the risk analysis of EV‐based biomarkers but also directly affect the detection of certain targets.

To overcome the difficulties in EV‐based studies, many efforts have been made by the to utilize varied technologies.^[^ ^50^ ^]^ A widely accepted criterion in regular EV studies was built by the MISEV2018, a very successful guideline for improving the comparability and repeatability of EV research.^[^ ^51^ ^]^ Nevertheless, the complex internality of new methodologies and technologies continuously presents new difficulties and challenges to this criterion.

## 3 Advanced Nanotechnologies for EV Isolation

Isolation is the first step in most EV studies. A series of advanced nanotechnologies have been developed for the isolation of EVs. To isolate EVs without any bias, physical feature‐based capture can be integrated with downstream functional assays or multiplex analysis. In addition, lipid probes, affinitive molecules, and TiO_2_ are important alternatives for nontargeted EV isolation. In contrast, targeted isolation can be implemented using specific molecules with high chemical affinity to EVs. These chemical affinity‐based EV isolation approaches largely simplify EV‐based liquid biopsy, but their capture efficiency still needs improvement. Thus, a proper combination of nanostructures and chemical affinity is proposed, with significantly higher efficiency and lower complexity of EV isolation.

### 3.1 EV Isolation Based on Physical Features

**Figure 2:**
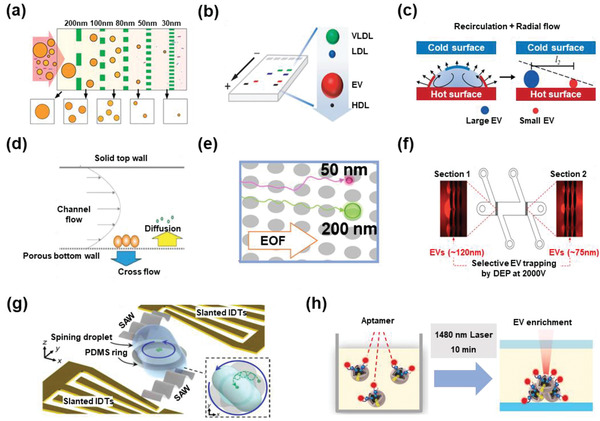
EV isolation based on physical features. a) Filtration. Reproduced with permission.^[^ ^52^ ^]^ Copyright 2017, American Chemical Society. b) Agarose gel‐based electrophoresis. Reproduced with permission.^[^ ^56^ ^]^ Copyright 2020, American Chemical Society. c) Marangoni flow based recirculation. Reproduced with permission.^[^ ^58^ ^]^ Copyright 2018, American Chemical Society. d) Asymmetric flow field‐flow fractionation. Reproduced with permission.^[^ ^60^ ^]^ Copyright 2019, Springer Nature. e) Electroosmotic flow driven‐deterministic lateral displacement. Reproduced with permission.^[^ ^64^ ^]^ Copyright 2019, American Chemical Society. f) Dielectrophoresis. Reproduced with permission.^[^ ^67^ ^]^ Copyright 2019, American Chemical Society. g) Acoustofluidic centrifugation. Reproduced with permission.^[^ ^72^ ^]^ Copyright 2021, American Association for the Advancement of Science. h) Thermophoresis. Reproduced with permission.^[^ ^73^ ^]^ Copyright 2019, Springer Nature.

The classical trichotomy of EVs relies on size distribution. Hence, a series of filtration‐based isolation strategies have been proposed and continuously optimized with delicate microfluidic designs (**Figure** **2**a).^[^ ^52^ ^]^ Usually, a polycarbonate membrane with 200–600 nm pores is used to block the entrance of large particles (e.g., cell debris) into downstream of microfluidic devices, and an anodic aluminum oxide or polycarbonate membrane with 30–50 nm pores is used to collect EVs.^[^ ^53^, ^54^ ^]^ Generally, series filtration is considered as the most common strategy for isolating EVs for materials scientists, and its validity has been widely proven. However, channel blockage resulted from two intractable congenital defects, that is, sized impurities and fluid shear force, largely preventing its widespread application.

Other size‐based EV isolation procedures, such as electrophoresis and Marangoni flow, have also been reported. Electrophoresis is a well‐established separation method invented in 1937, in which molecules exhibit different migration rates in an electric field owing to their different charge‐to‐size ratios.^[^ ^55^ ^]^ Generally, EVs are considered negatively charged nanoparticles. In 2020, Zhang et al. developed an agarose gel‐based electrophoresis technology to provide an effective approach for isolating EVs from plasma (Figure 2b).^[^ ^56^ ^]^ Size‐based EV isolation could also be achieved in a contact‐free manner. When locally affected by external disturbances (such as temperature and concentration), a surface tension gradient will be generated on the surface of the liquid, resulting in a recirculation flow, that is, Marangoni flow. For instance, Jeong et al. provided a spatial separation of EVs by taking advantage of Marangoni flow and the coffee‐ring effect in microdroplets (Figure 2c).^[^ ^57^, ^58^ ^]^ During the evaporation, the velocity of different nanoparticles changes from recirculation to radial flow along with a decrease in contact angles, and EVs of different sizes are aggregated and sorted by size after the drop dries out. This approach was tested by analyzing the subpopulations of plasma EVs. However, whether EVs can be directly isolated from other body fluids remains unknown.

EVs can also be separated based on their specific hydrodynamic characteristics. The asymmetric flow field‐flow fractionation (AF4) technology separates nanoscale soluble particles based on their density and hydrodynamic properties by allowing the sample to flow, perpendicularly.^[^ ^59^ ^]^ Additionally, Lyden's group further developed an AF4 protocol to fractionate EVs based on hydrodynamic sizes, showing a unique capability to separate nanoparticles with sizes ranging from 0.5 to 1000 nm (Figure 2d).^[^ ^60^ ^]^ Based on this approach, they identified a distinct EV subset (i.e., exomeres, ≈35 nm) with a smaller size compared to regular exosomes.^[^ ^38^ ^]^ Petersen et al. also demonstrated that cyclical electrical field flow fractionation, a technology similar to that of AF4, can be used to separate EVs.^[^ ^61^ ^]^ Generally, isolation approaches based on AF4 provide rapid, biocompatible, and label‐free isolation of EVs. However, the complicated fabrication and operation of these devices has largely limited their popularization in biomedical research.

The deterministic lateral displacement (DLD) approach is a classical method used for separating microscale particles based on pillar arrays, where micropillars are arranged at a certain angle to the direction of the flow. Particles of different sizes are isolated because large particles converge to one side, and small particles maintain their original straight trajectory. This approach has also been used to isolate EVs. For example, in 2016, Stolovitzky's team first implemented DLD approach to the nanoscale and separated particles of size between 20 and 110 nm.^[^ ^62^ ^]^ The same group further integrated DLD into a microfluidic platform for the separation of EVs.^[^ ^63^ ^]^ In 2019, Hattori et al. invented an electroosmotic flow‐driven DLD method to isolate EVs continuously (Figure 2e), which possessed an easy‐to‐operate flow control compared to the traditional DLD procedure.^[^ ^64^ ^]^ The DLD procedures showed good potential for size‐based EV isolation. However, other characteristics of EVs, such as density and stiffness, are non‐negligible interference factors in DLD‐based EV isolation.

Electrochemical technology can also be used for EV isolation. Nanoparticles can migrate in an asymmetrical electric field due to dielectric polarization, that is, dielectrophoretic (DEP) force, which can be used to selectively control or separate certain particles. For instance, in 2017, Heller's team invented a technique using an alternating current electrokinetic microarray chip to isolate EVs based on DEP separation force.^[^ ^65^ ^]^ They also integrated DEP isolation with subsequent immunofluorescent detection of specific protein biomarkers.^[^ ^66^ ^]^ Different PDMS‐based microfluidic designs and different materials used in DEP micropipettes have also been reported by other groups.^[^ ^67^, ^68^, ^69^ ^]^ In 2019, Moore et al. showed that binding of EVs to AuNP‐conjugated antibodies could modulate their conductance, and based on this phenomenon, they enabled selective microfluidic enrichment of EVs with different antigens (Figure 2f).^[^ ^67^ ^]^ Although DEP is a simple and promising EV separation approach, unwanted biological effects of DEP exerted on EVs would affect downstream analysis.

A surface acoustic wave (SAW) is an elastic mechanical wave that propagates along the surface of an elastic material, the amplitude of which attenuates exponentially with the depth of the surface. When the SAW exerts radiation forces on EVs, differential forces are generated to isolate different particles according to their mechanical properties (e.g., size, density, and compressibility). In 2015, Lee's group used an SAW‐based acoustic nanofilter to isolate EVs < 200 nm,^[^ ^70^ ^]^ and Wu et al. further integrated acoustic and microfluidic modules to isolate EVs from whole blood with a blood cell removal rate of over 99.999%.^[^ ^71^ ^]^ Huang's team developed an acoustofluidic centrifuge for nanoparticle enrichment and separation by entanglement of SAW actuation and the spin of a fluidic droplet (Figure 2g).^[^ ^72^ ^]^ Generally, SAW‐based acoustic nanofilters enable a simple contact­free method for EV isolation in a size‐tunable manner, but the farraginous cell debris and protein aggregates cannot be separated clearly.

Liquid phase‐based isolation of EVs, such as thermophoresis, is a very promising method owing to its homogeneous nature. The thermophoresis effect refers to the phenomenon that the nanoparticles migrate from the high‐temperature area to the low‐temperature area since the solvent molecules in the high‐temperature area, collide with nanoparticles with higher kinetic energy as compared to those in the low‐temperature area. Based on thermophoresis, Liu et al. proposed a novel EV capture and detection method with high detection sensitivity and an efficient reaction rate.^[^ ^73^ ^]^ After generating temperature gradients with a 1480 nm infrared laser, a size‐dependent isolation of EVs can be observed through the corresponding thermophoresis effect, diffusion, and convection (Figure 2h). The authors used this system to profile surface biomarkers of EVs,^[^ ^73^ ^]^ as well as to detect miRNAs inside EVs.^[^ ^74^ ^]^ This thermophoresis‐based system showed an ideal ability for the classification of pan‐cancerous patients and healthy individuals.^[^ ^73^, ^74^ ^]^ Additionally, thermophoresis‐based detection of EV‐derived PD‐L1 offers indicative information on early cancer diagnosis and immunotherapy response prediction.^[^ ^75^ ^]^ Besides thermophoresis, other liquid phase‐based detection methods have also been reported. For instance, Gao et al. reported an artificial Vir‐FV to detect EVs in the liquid phase in a membrane fusion manner.^[^ ^76^ ^]^ Liu et al. invented a droplet digital ExoELISA chip to capture EVs using magnetic microbeads, which constructed sandwich ELISA complexes tagged with an enzymatic reporter. By co‐encapsulating enzyme‐tagged microbeads and the substrate, the ExoELISA chip could generate a detectable fluorescent signal for EV counting.^[^ ^77^ ^]^ Thus, liquid phase‐based EV isolation could facilitate studies of EV biomarkers by providing a homogeneous basis for detection.

Without preselection of EV subpopulations, most physical feature‐based isolation nanotechnologies retain the primary bulk properties and biological activity of EVs. However, their clinical applications may be largely restricted because of the laborious fabrication of the facilities and the robustness of these complex systems. Thus, the simplification of the design and process technology should be considered in the subsequent development of these EV capture devices.

### 3.2 EV Isolation Based on Chemical Affinity

Molecules with chemical affinity to EVs have been widely explored in EV isolation. Generally, chemical affinity‐based EV isolation can be divided into two types: nontargeted and targeted EV capture. For nontargeted EV capture, EVs can be isolated using lipid probes, phosphatidylserine (PS) affinity molecules and TiO_2_ based on high affinity toward the lipids on the EV surfaces. For targeted EV capture, antibodies, aptamers, and peptides with high affinity toward various biomolecules on EV surfaces have been widely used.

**Figure 3:**
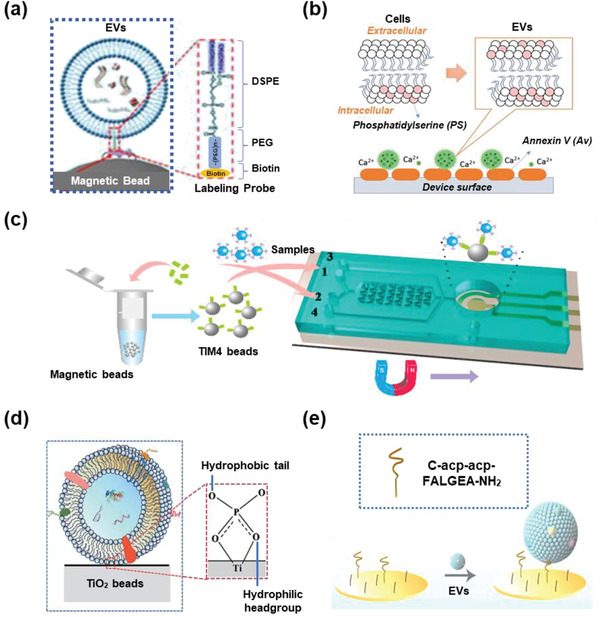
EV isolation based on chemical affinity. a) DSPE on a lipid probe affinity to EV membrane. Reproduced with permission.^[^ ^78^ ^]^ Copyright 2017, Springer Nature. b) Annexin A5 affinity to PS molecules. Reproduced with permission.^[^ ^83^ ^]^ Copyright 2019, Wiley‐VCH. c) TIM4 affinity to PS molecules. Reproduced with permission.^[^ ^85^ ^]^ Copyright 2018, American Chemical Society. d) TiO_2_ affinity to phosphate groups. Reproduced with permission.^[^ ^88^ ^]^ Copyright 2019, The Royal Society of Chemistry. e) Peptide affinity to EGFR. Reproduced with permission.^[^ ^95^ ^]^ Copyright 2020, American Chemical Society.

The lipid molecules on EV surfaces are considered important anchors in affinity‐mediated capture. Owing to the curvature of the membrane, EVs can transiently expose the hydrophobic core of the phospholipid to the aqueous phase, thus confirming the feasibility of capture by lipid probes. In 2017, Zheng's team designed and optimized a lipid nanoprobe system to label EVs for subsequent enrichment. The lipid nanoprobe consists of three functional parts: 1,2‐distearoyl‐*sn*‐glycero‐3‐phosphorylethanolamine (DSPE) to insert into the EV membrane due to a hydrophobic effect, PEG to provide solubility in the aqueous phase, and biotin to enable enrichment by avidin–biotin affinity (**Figure** **3**a).^[^ ^78^ ^]^ DSPE could be replaced with other lipophilic groups such as cholesterol to optimize its affinity,^[^ ^79^, ^80^ ^]^ while the adjustment of PEG spacers’ length could reduce steric hindrance and improve capture efficiency.^[^ ^79^ ^]^ An optimized cholesterol–PEG_1000_–biotin (≈6.4 nm length) was identified as the best lipid probe.^[^ ^79^ ^]^ Additionally, the lipid probe‐mediated membrane biotinylation strategy resulted in minimal damage to EVs and could be integrated with subsequent functional assays.^[^ ^81^, ^82^ ^]^

To maintain a stable nanosphere structure, the outer membranes of EVs were highly enriched in cone‐shaped lipids, such as PS molecules. Thus, molecules with high affinity to specific lipids have also been used in EV capture. Owing to its PS affinity, Annexin A5 has been widely used for the detection of cell apoptosis by flow cytometry. Compared to antibody coating, Annexin A5 coating largely improved the efficiency of EV isolation in ExoChip (Figure 3b).^[^ ^83^ ^]^ In addition, T‐cell membrane protein 4 (TIM‐4) is a natural receptor of the PS molecule, which has shown a higher affinity to PS molecules than Annexin A5 and other proteins.^[^ ^84^ ^]^ Hence, a TIM‐4‐based EV capture protocol was developed and tested for serum EV isolation in another study (Figure 3c).^[^ ^85^ ^]^ Other PS‐philic molecules, such as MFGE8,^[^ ^86^ ^]^ have also been identified and explored for their potential in future EV isolation studies. Another important feature of the PS molecule is its negative electricity. For instance, Chen et al. provided an efficient approach for the rapid isolation of EVs based on anion‐exchange chromatography.^[^ ^87^ ^]^

The phosphate groups on the surface of the EVs were also potential targets for EV capture. The hydrophilic surface of TiO_2_ can efficiently bind to these phosphate groups, which has been widely used for the enrichment of phosphorylated peptide. In 2019, Gao et al. captured EVs from serum based on specific interactions between the TiO_2_ microspheres and the phosphate groups on the EV surface (Figure 3d).^[^ ^88^ ^]^ Pang et al. also fabricated Fe_3_O_4_@TiO_2_ nanoparticles by hydrolysis reaction and obtained high efficiency for EV enrichment.^[^ ^89^ ^]^

Specific antibodies for targeting EV biomarkers, such as CD63, CD9, and CD81, are popular tools in EV capture, while antibodies targeting cancer‐specific membrane proteins, such as EpCAM, EGFR, and GPC‐1, are also frequently used in cancer‐derived EV isolation.^[^ ^90^, ^91^, ^92^ ^]^ It is worth noting that only antibodies that bind to the extracellular domain of EV membrane proteins are suitable for EV isolation. Antibodies used for EV isolation have already been summarized in other focused reviews.^[^ ^50^ ^]^

Aptamers are an alternative to antibodies for protein targeting. With a smaller size, aptamers can easily overcome the steric hindrance resulting from post‐translational modifications. For example, a new PD‐L1 aptamer developed by Yang's team showed a much lower dissociation constant compared to the PD‐L1 antibody, suggesting a higher affinity to PD‐L1.^[^ ^75^ ^]^ Aptamers binding to antigens such as CD63, EpCAM, PTK7, PSMA, PDGF, and HER2 have been applied in EV isolation.^[^ ^73^, ^93^, ^94^ ^]^ Additionally, aptamers are less likely to cause undesirable immune responses than antibodies, providing potential applicability for in vivo EV targeting.

Peptides with specific affinity to proteins on the surface of EVs have also been used for EV isolation. For instance, Sun et al. synthesized a peptide ligand for EGFR and modified gold electrodes with these peptides. Together with the Zr‐MOF‐based probes, this double recognition system enabled sensitive and label‐free detection of glioblastoma‐derived EVs from plasma (Figure 3e).^[^ ^95^ ^]^ Similarly, Zhao et al. used three different tumor antigenic peptides (gp‐100, MAGE‐A3, and MART‐1) to enrich MHC‐I positive, antigenic EVs produced by leukocytes. Interestingly, both gp‐100 and MART‐1 showed an advantage in MHC‐I + EV capture over the MHC‐I antibody, while the gp‐100 peptide exhibited the highest efficiency.^[^ ^96^ ^]^

In summary, nontargeted capture tools are widely used for EV isolation, such as lipid probes, PS affinity molecules, and TiO_2_. Other molecules on the EV surfaces are also targetable by specific antibodies, aptamers, and peptides in targeted EV isolation. Therefore, we believe that more approaches would be developed for chemical affinity‐mediated EV capture with a much deeper understanding of EV chemical compositions and EV biological characteristics.

### 3.3 EV Isolation Based on the Combination of Nanostructures and Chemical Affinity

Even though chemical affinity‐based EV isolation greatly promotes EV‐based liquid biopsy, there is still a long way to go before making these approaches clinically applicable. The involvement of proper nanostructured surfaces in EV isolation would not only provide a higher efficient reaction rate but also overcome interfacial EV bindings, boundary effects, and limits to microscale mass transfer. Therefore, the combination of nanostructures and chemical affinity is a new paradigm for high‐efficiency EV isolation.

**Figure 4:**
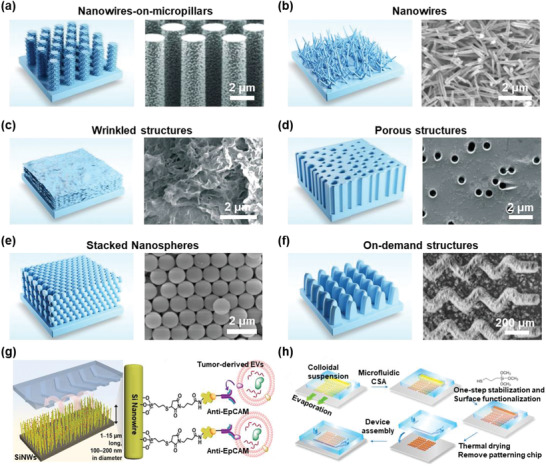
EV isolation based on the combination of nanostructures and chemical affinity. a–f) The schematic diagrams were displayed on the left panels, while the representative SEM images were shown on the right panels. a) Nanowire‐on‐micropillars. Reproduced with permission.^[^ ^97^ ^]^ Copyright 2013, The Royal Society of Chemistry. b) Nanowires. Reproduced with permission.^[^ ^98^ ^]^ Copyright 2017, American Association for the Advancement of Science. c) Wrinkled structures. Reproduced with permission.^[^ ^99^ ^]^ Copyright 2016, The Royal Society of Chemistry. d) Porous structures. Reproduced with permission.^[^ ^101^ ^]^ Copyright 2017, American Chemical Society. e) Stacked nanospheres. Reproduced with permission.^[^ ^90^ ^]^ Copyright 2019, The Royal Society of Chemistry. f) On‐demand structures. Reproduced with permission.^[^ ^103^ ^]^ Copyright 2020, American Association for the Advancement of Science. g) Anti‐EpCAM modified Si nanowire in a microfluidic chaotic mixer. Reproduced with permission.^[^ ^106^ ^]^ Copyright 2019, American Chemical Society. h) Anti‐CD81 coated SiO_2_ nanostructures. Reproduced with permission.^[^ ^109^ ^]^ Copyright 2019, Springer Nature.

Micro/nanoscale interfacial materials with designed structures have been developed for EV capture, including nanowire‐on‐micropillars, nanowires, wrinkled structures, porous structures, stacked nanospheres, and on‐demand structures. For instance, Wang et al. fabricated a porous silicon nanowire‐on‐micropillar structure using electroless metal‐assisted nanowire etching. EVs could be effectively trapped by the interstitial sites between the ciliated micropillars (**Figure** **4**a).^[^ ^97^ ^]^ Silicon nanowires are another popular structure for EV isolation. For example, Yasui et al. developed a device containing a nanowire‐embedded PDMS substrate that enabled the electrostatic collection of urinary EVs (Figure 4b).^[^ ^98^ ^]^

Graphene‐based materials were usually used in the synthesis of wrinkled nanostructures. For instance, Zhang et al. developed a microfluidic device with a highly wrinkled graphene oxide/polydopamine (GO/PDA) interface, which could be easily modified by different antibodies, enabling targeted EV capture (Figure 4c).^[^ ^99^ ^]^ Similarly, Fang et al. took advantage of the highly wrinkled structure of PDA‐functionalized graphene foam in EV capture.^[^ ^100^ ^]^

Porous structures from various materials can be used for EV capture. For example, the porous ExoTENPO device was fabricated by thermally evaporating a 200 nm Ni_80_Fe_20_ permalloy layer (Figure 4d).^[^ ^101^ ^]^ Han et al. developed a Morpho butterfly wing‐integrated microvortex biochip with microscale‐sized grooves to increase the interaction between EVs and lipid probes.^[^ ^102^ ^]^

Notably, various nanointerfaces can be customized from nanostructured SiO_2_, such as colloidal silica. In Figure 4e,f, Zeng's group obtained various nanointerfaces by microfluidic engineering, such as stacked nanospheres and on‐demand structures.^[^ ^90^, ^103^ ^]^ In addition, synthetic materials such as poly(amidoamine) (PAMAM) dendrimers have been utilized to provide sufficient steric contact with EVs. By utilizing multivalent binding effects of PAMAM dendrimers, Zhang et al.^[^ ^104^ ^]^ and Poellmann et al.^[^ ^105^ ^]^ captured EVs on a PAMAM dendrimer‐coated surface. In summary, various nanostructures have been developed for advanced EV capture nanotechnologies, which simultaneously enable high affinity to EVs and an increased contact chance between EVs and the capture interface.

The preparation technologies and manufacturing strategies of functionalized nanoscale materials for isolating EVs have improved rapidly. To specifically isolate cancer cell‐derived EVs from the blood, Dong et al. developed a Si nanowire chip by ion etching and decorated the surface of the nanowires with EpCAM antibodies (Figure 4g).^[^ ^106^ ^]^ Similarly, Zhu and Tseng's team further developed a specific EV purification system by integrating covalent chemistry‐mediated EV capture on a Si nanowire‐based microfluidic chaotic mixer, enabling various downstream functional studies.^[^ ^107^, ^108^ ^]^ The colloidal self‐assembly (CSA) of nano SiO_2_ is a promising approach to improve the specific surface area of the materials and to create different functionalized nanoscale interfaces. Zeng's team fabricated serpentine nanostructures on a bead pattern chip sealed by nano SiO_2_ CSA and adopted a divarication microfluidic design (ExoProfile) integrated with various antibodies to enable multiplexed detection of biomarkers on EVs.^[^ ^90^ ^]^ Recently, they further carried out a multiscale integration by designing a self‐assembly strategy to obtain various large‐scale 3D nanostructured elements by engineered CSA in microfluidics, exhibiting ultrasensitive detection ability of cancer‐derived EVs (Figure 4h).^[^ ^109^ ^]^ They also tried an open‐surface 3D printing technology to improve the scalability and success rate of fabrication.^[^ ^103^ ^]^ In addition, molecular imprinting is another popular approach for bioseparation. For example, Mori et al. created EV‐shaped cavities in polymer matrices with predetermined selectivity provided by conjugating antibodies on the surfaces of those cavities,^[^ ^110^ ^]^ and this molecular imprinting strategy showed good performance in identifying breast cancer patients from normal individuals by analyzing EVs from tears.^[^ ^111^ ^]^

The combination of nanostructures and chemical affinity is a common strategy to improve EV capture, which originates from the synergistic effect of structural matching and molecular recognition. Nonetheless, further studies are required to simplify the fabrication process of EV capture interfaces and improve the specific isolation of different EVs.

## 4 Advanced Nanotechnologies for EV Detection

The low concentration of EVs has led to an urgent demand for highly sensitive detection technologies. Recently, the limit of detection (LoD) of EVs has been largely reduced by utilizing advanced sensors such as optical sensors, electrochemical sensors, and other sensors. Furthermore, single EV detection has been developed to fully address the heterogeneity of EVs.

### 4.1 Optical Sensors

The optical sensors used in EV detection can be roughly divided into fluorescent and spectral signals. To visualize and quantify EVs or their surface biomarkers, fluorescent signals are detected by linking fluorescent probes with antibodies or aptamers. To improve the LoD of EVs, different generation modes of fluorescent signals, such as fluorescence quenching, molecular beacons, quantum dots (QDs), and fluorescence polarization, were adopted. In addition to the direct detection of fluorescent signals, spectral signal sensing is a good alternative for EV detection, including near‐infrared (NIR), surface plasmon resonance (SPR), and surface‐enhanced Raman spectroscopy (SERS).

**Figure 5:**
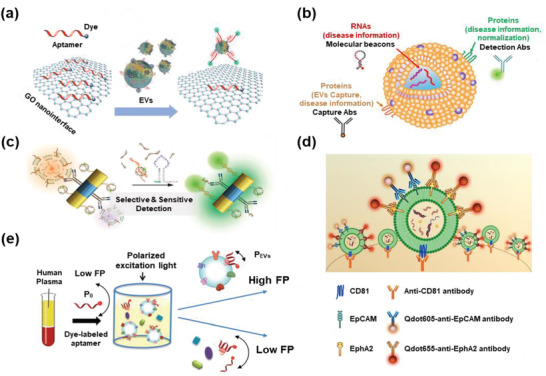
EV detection based on fluorescent signals of optical sensors. a) GO quenching. Reproduced with permission.^[^ ^93^ ^]^ Copyright 2018, American Chemical Society. b) Molecular beacons. Reproduced with permission.^[^ ^114^ ^]^ Copyright 2019, Elsevier. c) Metal‐enhanced fluorescence. Reproduced with permission.^[^ ^115^ ^]^ Copyright 2019, American Chemical Society. d) Quantum dots (QDs). Reproduced with permission.^[^ ^117^ ^]^ Copyright 2019, American Chemical Society. e) Fluorescence polarization. Reproduced with permission.^[^ ^120^ ^]^ Copyright 2019, The Royal Society of Chemistry.

Fluorescence quenching is widely used to obtain more sensitive detection of EVs than direct fluorescent imaging. GO is the first reported 2D material with fluorescence quenching ability. The *π*–*π* stacking interaction strongly binds the aptamers on the surface of GO, and the fluorescence is quenched by fluorescence resonance energy transfer between the conjugated dyes and GO. For instance, Zhang's group quenched FAM‐labeled aptamers on GO membranes, and EVs with specific surface proteins would competitively bind to these aptamers and exhibit fluorescent signals with an LoD of 160 EVs µL^−1^ (**Figure** **5**a).^[^ ^93^ ^]^ Oh et al. quenched the FAM‐labeled miR‐193a probes with GO and successfully visualized miR‐193a in EVs on a microfluidic platform.^[^ ^112^ ^]^ Tayebi et al. developed a fluorometric nanosensor based on MoS_2_ multiwall carbon nanotubes (MWCNTs), which quenched phycoerythrin‐conjugated CD63 antibodies. The fluorescent antibodies would detach from MWCNTs when they bind to EVs, providing an LoD of 1480 EVs µL^−1^.^[^ ^113^ ^]^

In addition to fluorescence quenching based on carbon nanostructures, nucleic acid base pairing can also control fluorescent signals. A molecular beacon is a hairpin‐shaped single‐stranded DNA with an internally quenched fluorophore. The fluorophore is released and the fluorescence is restored when the molecular beacon binds to a complementary sequence, such as an EV‐derived miRNA. For example, Cho et al. designed a series of miRNA molecular beacons that could detect EV‐derived miRNAs and surface proteins simultaneously in combination with antibodies (Figure 5b).^[^ ^114^ ^]^ Gao et al. engineered a virus‐mimicking fusogenic vesicle (Vir‐FV) for rapid detection of EV‐derived microRNAs. The virus fusogenic proteins on Vir‐FVs specifically targeted EVs via sialic acid‐containing receptors and promoted vesicle fusion. The self‐paired and quenched molecular beacon could specifically bind to miR‐21 and become visible, providing a miR‐21 LoD of 1.3 × 10^−9^ m.^[^ ^76^ ^]^

Fluorescent signals can be amplified by metal‐enhanced fluorescence (MEF). For example, Lee et al. reported an antibody‐conjugated multifunctional magneto‐plasmonic nanorod. EVs could be captured by the magnetic nanorods with antibody affinity, and the released miRNAs could be detected by the molecular beacons binding to the nanorods. The MEF effects of the Au plasmon could further amplify the fluorescent signals (Figure 5c).^[^ ^115^ ^]^ This MEF system was used to analyze EV‐derived miRNAs in a conditioned medium to control stem cell differentiation, and its potential in detecting EV biomarkers derived from human body fluids requires further investigation.

QDs are inorganic nanocrystals with unique optical properties. Compared to common fluorescent dyes, QDs show a broad and intense absorption, enabling unique flexibility in excitation and a higher fluorescence quantum yield.^[^ ^116^ ^]^ Rodrigues et al. labeled a series of antibodies with different QDs and quantified the multiplex EV surface proteins (Figure 5d). The new method showed an improved ability to detect two pancreatic cancer associated EV biomarkers (EpCAM and EphA2).^[^ ^117^ ^]^ Tan's group synthesized a self‐powered TiO_2_@MoS_2_ QD‐based probe, which enabled ultrasensitive detection of EV‐derived HOTTIP RNA with an LoD of 5 fg mL^−1^.^[^ ^118^ ^]^ With unique optical properties, the QD signal can be easily enhanced. For example, Zhang's team designed a diagnostic biochip to detect EVs by combining the advantages of QDs with the biomimetic periodic nanostructure of photonic crystals.^[^ ^119^ ^]^

A fluorescence polarization assay was also designed to detect EVs, in which the inherent high mass/volume of EVs acts as a mass‐based fluorescence polarization amplifier. Thus, dye‐labeled aptamers binding to EVs generate a higher signal than free dye‐labeled aptamers. Fang's research group quantified EVs using dye‐labeled aptamers, showing a higher fluorescence polarization than free‐floating aptamers (Figure 5e).^[^ ^120^ ^]^ This fluorescence polarization‐based quantification exhibited an LoD of 500 EVs µL^−1^ in the range of 500–500 000 EVs µL^−1^.

**Figure 6:**
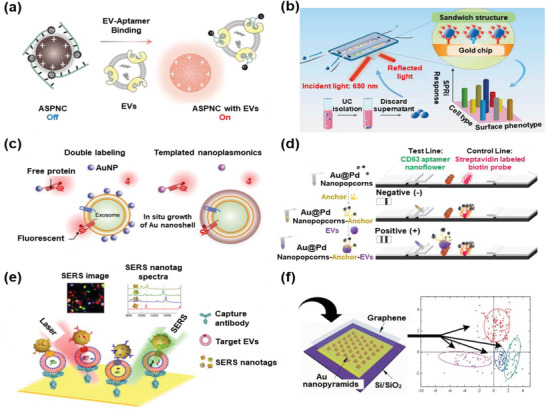
EV detection based on spectral signals of optical sensors. a) Afterglow signals in NIR. Reproduced with permission.^[^ ^121^ ^]^ Copyright 2019, Wiley‐VCH. b) Surface plasmon resonance (SPR) signals. Reproduced with permission.^[^ ^122^ ^]^ Copyright 2020, Elsevier. c) Redshift signals in SPR. Reproduced with permission.^[^ ^125^ ^]^ Copyright 2020, American Association for the Advancement of Science. d) Photothermal signals in a lateral flow assay. Reproduced with permission.^[^ ^127^ ^]^ Copyright 2019, American Chemical Society. e) Surface‐enhanced Raman spectroscopy (SERS) nanotag spectral signals. Reproduced with permission.^[^ ^132^ ^]^ Copyright 2020, American Association for the Advancement of Science. f) Label‐free SERS spectral signals. Reproduced with permission.^[^ ^136^ ^]^ Copyright 2019, American Chemical Society.

Compared to common fluorescence, spectral signals afford a much lower background and better LoD. For instance, Lyu et al. developed an NIR afterglow luminescent nanosensor by adding quencher‐tagged aptamers to an NIR semiconducting polyelectrolyte complex. The binding of targeted EVs to aptamers would increase the distance between the polyelectrolyte complex and quencher and turn on the afterglow signal (**Figure** **6**a).^[^ ^121^ ^]^ This system exhibited an LoD of 0.24 ng µL^−1^ in both PBS and plasma environments, enabling accurate identification of the cellular origin of circulating EVs for cancer diagnosis. SPR refers to the resonant oscillation of conduction electrons stimulated by polarized light at the interface of media with different refractive indices. SPR signal detection is another sensitive label‐free technology that can detect molecular interactions occurring on an Ag/Au surface based on monitoring changes in the refractive index resulting from binding.^[^ ^91^ ^]^ Fan et al. established an SPR imaging biosensing assay for highly sensitive and multiplex detection of lung cancer cell‐derived EVs (Figure 6b).^[^ ^122^ ^]^ There were several designs to improve the sensitivity of SPR sensors. Wang et al. used dual gold nanoparticle (AuNP)‐assisted signal amplification in an SPR imaging system and achieved an LoD of 5 EVs µL^−1^.^[^ ^123^ ^]^ Similarly, Liu's group constructed an Au nanocluster membrane–EV–Au nanorod complex, which generated an improved scattering intensity due to resonance Rayleigh scattering, and further improved the LoD to 1 EVs µL^−1^.^[^ ^124^ ^]^

Nanoscale Au particles exhibit a redshift in their plasmonic absorbance spectra when their size increases. For instance, Wu et al. enabled in situ growth of Au nanoshells on EVs, resulting in a large redshift in Au‐based plasmonic resonance and quenching of fluorescent probes targeted to EV membrane proteins (Figure 6c).^[^ ^125^ ^]^ This system exhibited an LoD of ≈1500 EVs and was utilized in the prognosis of both gastric and colorectal cancers. The AuNP‐based lateral flow assay (LFA) is a cost‐effective and sensitive method for detecting EVs based on colorimetry.^[^ ^126^ ^]^ Lin et al. integrated Au@Pd nanosphere redshift effect sensors with aptamer nanoclusters for EV capture on an LFA strip and enabled a thermal signal readout (Figure 6d).^[^ ^127^ ^]^ The Au@Pd nanosphere and aptamer nanocluster‐assisted lateral flow strip device can detect EVs from serum and exhibit a balance between sensitivity and portability.

The interaction between molecules with a nanostructured metal surface would amplifies their Raman scattering signal, which is widely used to identify chemical species and EV detection, that is, SERS sensors. For instance, Stremersch et al. deposited an AuNP‐based shell on the surface of EVs, which enhanced the Raman signal while maintaining a colloidal suspension of individual EVs.^[^ ^128^ ^]^ Furthermore, they improved the efficiency of core−shell (Au@Ag) nanoparticles (NPs) as a SERS substrate and obtained a higher near‐field enhancement within a 1/20 EV acquisition time as compared to the previous version.^[^ ^129^ ^]^ Moreover, Li et al. developed a novel SERS probe, that is, ultrathin polydopamine‐encapsulated antibody‐reporter‐Au@Ag multilayer, using which ultrasensitive detection of cancer cell EVs with an LoD of 0.5 EVs mL^−1^ was possible.^[^ ^130^ ^]^ Dong et al. fabricated a beehive‐inspired Au‐coated TiO_2_ macroporous inverse opal structure with an outstanding SERS effect to detect EVs from human plasma.^[^ ^131^ ^]^ In addition, various SERS tags can be used to enable multiplex detection of EV biomarkers. For instance, Wang et al. developed a multiplex EV phenotype analyzer chip by integrating a nanomixing‐enhanced microchip with multiplex SERS nanotags (Figure 6e). This system showed promising applicability in the treatment monitoring of patients with melanoma.^[^ ^132^ ^]^ Similarly, Zhang et al. developed three antibody‐conjugated SERS probes with different spectral emissions, enabling multiplex EV surface protein detection in a mimetic plasma sample.^[^ ^133^ ^]^

Moreover, general SERS signals were collected to reflect the physicochemical properties of the captured label‐free EVs. For example, Lee et al. used thin silver film‐coated SERS substrates with bowl shapes to capture EVs and proved that the SERS signals could provide a biochemical analysis of intact and ruptured EVs.^[^ ^134^ ^]^ Fortunato's group fabricated a bacterial nanocellulose from commercial Nata de coco and in situ synthesized silver NPs to detect EVs,^[^ ^135^ ^]^ while Yan et al. fabricated a graphene‐covered Au surface containing a quasi‐periodic array of pyramids as SERS substrates for EV detection (Figure 6f).^[^ ^136^ ^]^ Both these devices could successfully distinguish EVs with different cell origins. Furthermore, Choi's team recorded the SERS spectra of EVs secreted from different cells and trained a Resnet‐based deep learning model based on these data. This ad hoc model could predict lung cancer patients by detecting the SERS spectrum of their plasma EVs with an AUC of 0.912.^[^ ^137^ ^]^

In summary, optical sensor‐based detection provides direct observation of EVs derived from body fluids. The full‐fledged signal reception and processing devices for fluorescent signals make these techniques much closer to clinical applications. Advanced fluorescence technologies and spectral sensors, such as MEF, QDs, SPR, and SERS, have been applied to improve the sensitivity. However, many of these LoDs were evaluated in mimetic blood samples, and the actual abilities of these technologies in detecting EVs from real body fluids should be interpreted with caution. Additionally, the signal reception and processing devices for advanced optical sensors are not available in most biomedical laboratories, which may limit their further applications.

### 4.2 Electrochemical Sensors

Horseradish peroxidase (HRP)‐based colorimetry and electrochemical luminescence (ECL) have been widely used for EV detection with a 3,3′′,5,5′′‐tetramethylbenzidine (TMB) substrate. For example, Lee's team developed a paper‐based ELISA for EV isolation and detection using an HRP‐conjugated CD9 antibody.^[^ ^138^ ^]^ Improved performance can be achieved by ultrasensitive nanoprobes, including Ti_3_C_2_ MXene, ferrocene (Fc), and metal ions. To further improve the LoD of EVs, dual signal detection systems, such as CG‐quadruplex/Hemin and nano‐interdigitated electrodes, have also been explored.

**Figure 7:**
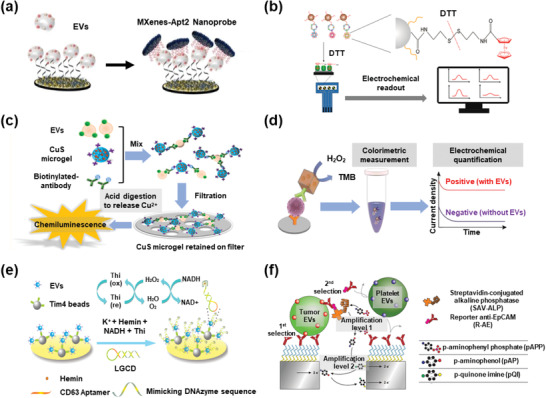
EV detection based on electrochemical sensors. a) Ti_3_C_2_ MXenes enhanced electrochemical signals. Reproduced with permission.^[^ ^139^ ^]^ Copyright 2019, Elsevier. b) Ferrocene‐enhanced electrochemical signals. Reproduced with permission.^[^ ^142^ ^]^ Copyright 2020, American Chemical Society. c) Cu^2+^ ion released by CuS‐Micogel produced chemiluminescence. Reproduced with permission.^[^ ^143^ ^]^ Copyright 2019, American Chemical Society. d) Peroxidase‐like activity of NPFe_2_O_3_N induced electrochemical signals. Reproduced with permission.^[^ ^144^ ^]^ Copyright 2019, American Chemical Society. e) Hemin/G‐quadruplex aptasensors with both NADH oxidase activity and HRP‐mimicking DNAzyme activity. Reproduced with permission.^[^ ^85^ ^]^ Copyright 2018, American Chemical Society. f) Nano‐interdigitated electrodes to detect electrochemical signals. Reproduced with permission.^[^ ^147^ ^]^ Copyright 2020, American Chemical Society.

Aptamer‐conjugated Ti_3_C_2_ MXenes have been used as alternative HRP sensors for EV detection owing to their large surface area and excellent catalytic activity. For example, Zhang et al. enabled EV detection on an aptamer‐modified Au surface using Ti_3_C_2_ MXene‐aptamer‐based nanoprobes with an LoD of 125 EVs µL^−1^ in a mimetic serum sample (**Figure** **7**a).^[^ ^139^ ^]^ A further improved LoD of 30 EVs µL^−1^ was obtained by using AuNP‐enhanced Ti_3_C_2_ MXenes for EV detection.^[^ ^140^ ^]^

Fc is another ultrasensitive HRP nanoprobe used for EV detection. For instance, An et al. used CD63 aptamer‐modified microbeads and CD63 aptamer‐conjugated Fc to isolate and tag EVs. The link between Fc and aptamers could be broken by 1,4‐dithiothreitol treatment, and the HRP activity of Fc finally enabled sensitive EV detection (Figure 7b). ^[^ ^141^ ^]^ Sun et al. doped Fc molecules onto a ZIF‐67/ITO film by electrodeposition. Additional black phosphorus nanosheets were cast on this film to immobilize methylene blue (MB)‐labeled aptamers. The Fc signal‐calibrated MB signal could further achieve an LoD of 0.1 EVs µL^−1^.^[^ ^142^ ^]^

Various metal ions with HRP activity (e.g., Cu^2+^, Fe^3+^, and Ru(bpy)_3_^2+^ ions) were also used for EV detection. For instance, Jiang et al. captured EVs using antibody‐conjugated CuS‐enclosed microgels. The Cu^2+^ ions could be released by acid digestion, generating a strong chemiluminescence signal, enabling an LoD of 10 EVs µL^−1^ (Figure 7c).^[^ ^143^ ^]^ Chen et al. isolated EVs using anion‐exchange magnetic beads and detected them using aptamer‐capped Fe_3_O_4_ NPs. The EVs competitively bound to aptamers and reduced the peroxidase activity of Fe_3_O_4_ NPs, enabling an LoD of 3580 EVs µL^−1^.^[^ ^87^ ^]^ Boriachek et al. developed a gold‐loaded ferric oxide nanocube with high peroxidase activity and achieved a LoD of 1 EVs µL^−1^ based on colorimetry (Figure 7d).^[^ ^144^ ^]^ Other substrates, instead of TMB, were also tested for metal ion‐based HRP signal detection. For instance, Fan et al. captured EVs using magnetic beads and quantified miR‐21 in EVs with a Ru(bpy)_3_^2+^‐polymer‐amplified ECL strategy based on tripropylamine.^[^ ^145^ ^]^

The hemin/G‐quadruplex system is a popular nanosensor with both DNAzyme and NADH oxidase activity for EV detection. For example, Ye's team developed an on‐chip EV detection system with a hemin/G‐quadruplex nanosensor, enabling an LoD of 4.39 EVs µL^−1^ (Figure 7e).^[^ ^85^ ^]^ Li's group developed a dual signal amplification strategy by combining hemin/G‐quadruplex‐assisted signal amplification with linker DNA amplification to detect EVs. This system exhibited high selectivity and sensitivity toward cancer‐derived EVs with an LoD of 0.954 EVs µL^−1^.^[^ ^146^ ^]^ Other dual signal amplification strategies have also been tested for EV detection. Mathew et al. designed a sandwich immunoassay on antibody‐modified nanoelectrodes by taking advantage of an enzymatic assay (catalyzing *p*‐aminophenyl phosphate to *p*‐aminophenol) and a redox cycling on nano‐interdigitated electrodes (catalyzing *p*‐aminophenol into *p*‐quinone imine) in signal amplification (Figure 7f),^[^ ^147^ ^]^ and obtained an LoD of 5 EVs µL^−1^ with an excellent linear response covering six orders of magnitude.

Therefore, electrochemical sensors are widely used in EV detection. Colorimetric signals can be easily evaluated with a microplate reader or even with the naked eye, whereas electrical signals are more precise and easier to be integrated with electronics. As ultrasensitive nanoprobes, novel catalysts and substrates are continually emerging, EV detection based on integrated electrochemical signals is an important and promising direction for liquid biopsy.

### 4.3 Other Sensors

In addition to optical and electrochemical sensors, EVs in body fluids can be detected by employing other sensors such as field‐effect transistor (FET) sensors, magnetic sensors, and mechanical sensors.

**Figure 8:**
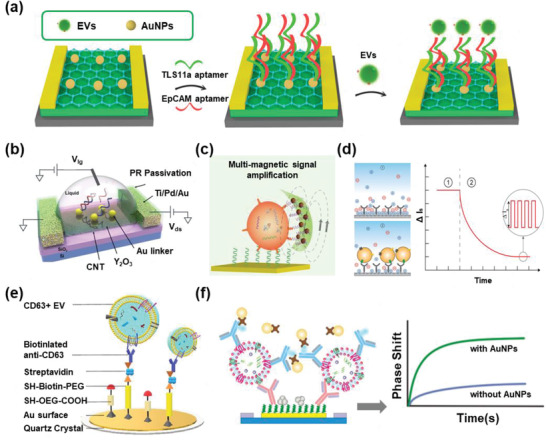
EV detection based on other sensors. a) Electrical signals from rGO field‐effect transistors (FETs). Reproduced with permission.^[^ ^148^ ^]^ Copyright 2020, American Chemical Society. b) Electrical signals from CNT FETs. Reproduced with permission.^[^ ^150^ ^]^ Copyright 2020, American Chemical Society. c) Giant magnetoresistance signals from a 2D MoS_2_–Fe_3_O_4_ hybrid nanostructure (MOFE) probe. Reproduced with permission.^[^ ^151^ ^]^ Copyright 2020, Elsevier. d) Electrokinetic signals from EVs captured on a silica microcapillary. Reproduced with permission.^[^ ^152^ ^]^ Copyright 2019, American Chemical Society. e) Dissipation signals from EVs captured on quartz crystal microbalance. Reproduced with permission.^[^ ^153^ ^]^ Copyright 2020, American Chemical Society. f) Surface acoustic wave (SAW) signals from AuNPs targeted EVs. Reproduced with permission.^[^ ^154^ ^]^ Copyright 2020, American Chemical Society.

FET sensors have been widely used for high‐sensitivity EV sensing. The shape of the conductive channel could be controlled by the electric field, resulting in a change in conductivity, that is, the FET effect. Biological signals can be transferred to electrical signals with high sensitivity by exploiting this effect. For instance, Wu et al. reported a highly sensitive detection of EVs using a reduced graphene oxide (rGO)‐based FET biosensor. They captured EVs with a DSPE lipid probe and quantified them using FET‐based detection with an LoD of 20 EVs µL^−1^.^[^ ^81^ ^]^ Additionally, an AuNP‐decorated, dual aptamer‐modified rGO FET nanosensor was developed for label‐free and sensitive quantification of plasma EVs (**Figure** **8**a).^[^ ^148^ ^]^ FETs can also be used for the detection of EV‐derived miRNAs. Cheng et al. captured EVs in an integrated microfluidic chip with an FET sensing module and achieved a promising LoD (6.069 × 10^−15^ m) of EV‐derived miR‐21.^[^ ^149^ ^]^ Carbon nanotubes (CNTs) are considered to be ideal materials for the fabrication of FET‐based biosensors. Liang et al. added a 6 nm Y_2_O_3_ high‐*κ* dielectric layer as a floating gate structure on the CNT films, which further improved the sensitivity of the CNT FET‐based sensor to 6 particles mL^−1^ for EV detection (Figure 8b).^[^ ^150^ ^]^

Magnetic sensors are promising alternatives for EV detection. For instance, Zhu et al. designed a 2D magnetic MoS_2_–Fe_3_O_4_ hybrid nanostructure (MOFE) as an ultrasensitive giant magnetoresistance (GMR) sensor for probing plasma EVs (Figure 8c).^[^ ^151^ ^]^ The aptamer‐conjugated MOFE detected a limit of 100 EVs, which could be used to distinguish patients with ovarian cancer from healthy individuals. Electrokinetic sensors have also been used for the detection of EVs. For example, Cavallaro et al. developed an electrokinetic sensor on the inner surface of a silica microcapillary and enabled EV detection (Figure 8d).^[^ ^152^ ^]^ Mechanical signal detection has also been applied to EV‐based liquid biopsies. For instance, Guldin's team applied quartz crystal microbalance with dissipation monitoring to detect EVs and obtained an LoD of 1.4 × 10^5^ EVs µL^−1^ (Figure 8e).^[^ ^153^ ^]^ As we previously introduced, many groups isolated different EV subsets using the SAW force. Moreover, the same element used to generate SAWs could also be used for EV detection. With an AuNP‐based amplification of the SAW signal and a sensitive SAW sensor, Zhang's team obtained an LoD of 1100 EVs µL^−1^ (Figure 8f).^[^ ^154^ ^]^

Together, these studies highlighted the performance of various sensors in EV detection and reported promising LoDs for many new approaches (**Table** **1**). However, the comprehensive and expensive signal‐processing devices largely limit their further application. Additionally, these studies were based on various body fluids and different isolation technologies, suggesting that further parallel comparisons with strictly controlled conditions are needed to provide a fair assessment.

**Table 1:** Recent advanced nanotechnologies in detecting EVs from body fluids

Disease	Clinical application	Body fluids	Isolation technology	Detection technology	LoD	Participants No.	Ref.
BRCA^a)^	Diagnosis	Plasma	Antibody‐modified MBs	FL based DNA TWJs cyclic amplification	65 000 EVs µL^−1^	37BRCA + 10NC	^[^ ^155^ ^]^
BRCA	Diagnosis	Plasma	Aptamer‐modified BPNSs/Fc/ZIF‐67/ITO nanostructures	Fc based redox potentials	0.1 EVs µL^−1^	4BRCA + 3NC	^[^ ^142^ ^]^
BRCA	Diagnosis	Plasma	Aptamer‐modified MBs	3D‐DNA walking amplification and ExoIII based redox signals	13 EVs µL^−1^	3BRCA + 3NC	^[^ ^156^ ^]^
BRCA	Diagnosis	Plasma	Antibody and droplet microfluidic	Droplet digital ExoELISA	10 EVs µL^−1^	12BRCA + 10NC	^[^ ^77^ ^]^
BRCA	Diagnosis	Serum	Antibody‐modified MBs	Fc based redox signals	NA	4BRCA + 4NC	^[^ ^141^ ^]^
BRCA	Diagnosis	Serum	Filtration based chip	Aptamer concentration difference	8.9 EVs µL^−1^	6BRCA + 7NC	^[^ ^54^ ^]^
BRCA	Diagnosis	Serum	rVir membrane fusion	MBs based FL	1.3 × 10^−9^ m (miR‐21)	5Cancer + 5NC	^[^ ^76^ ^]^
BRCA	Diagnosis	Serum	SEC	HB pencil core‐based impedance spectrum	NA	8BRCA + 4NC	^[^ ^157^ ^]^
BRCA	Diagnosis	Serum	Aptamer‐modified MBs	Lipid probe‐conjugated DNA HCR amplification and Au‐Au@Ag NPs colometry	160 EVs µL^−1^	3BRCA + 2NC	^[^ ^158^ ^]^
BRCA	Diagnosis	Serum	Aptamers thermophoresis	Nanoflare based FL	0.36 × 10^−15^ m (miR‐375)	17BRCA + 12NC	^[^ ^74^ ^]^
BRCA	Diagnosis	Tear	Molecular imprint	FL labeled antibodies	7.224 EVs µL^−1^	5BRCA + 5NC	^[^ ^111^ ^]^
BRCA	Diagnosis and Staging	Plasma	Antibody‐modified SiO_2_ nanostructure	FRET probes	16 EVs µL^−1^	54BRCA + 46NC	^[^ ^103^ ^]^
BRCA	Diagnosis and Staging	Serum	Antibody‐modified MBs	Antibody‐conjugated DNA RCA amplification and PVP@HRP@ZIF‐8	0.334 EVs µL^−1^	15BRCA + 6NC	^[^ ^159^ ^]^
GBM	Diagnosis	Serum	EGFR (EGFR vIII) affinity peptide‐modified Au surface	MB@UiO‐66 (Zr‐MOFs)	9500 EVs µL^−1^	16GBM + 8NC	^[^ ^88^ ^]^
GC	Diagnosis	Plasma	Filtration and Aptamers	Aptamer based RCA amplification	42.7 EVs µL^−1^	10GC + 12NC	^[^ ^160^ ^]^
HCC	Diagnosis	Serum	TIM4‐modified PDMS chip	G‐quadruplex/Hemin based HRP reporter	4.39 EVs µL^−1^	10HCC + 6NC	^[^ ^85^ ^]^
HCC	Diagnosis	Serum	Aptamer‐modified Au surface	rGO based FETs	84 EVs µL^−1^	10HCC + 10NC	^[^ ^148^ ^]^
HNSCC	Diagnosis	Plasma	Antibody‐modified G7‐PAMAM dendrimers nanostructure	AFM	NA	5HNSCC + 8NC	^[^ ^105^ ^]^
LC	Diagnosis	Plasma	Microchambers separation	Multi‐color qPCR	10 copies µL^−1^	32LC + 30NC	^[^ ^161^ ^]^
LC	Diagnosis	Plasma	Fe_3_O_4_@TiO_2_ affinity	SERS nanotags	1 EVs µL^−1^	7LC + 12 NC	^[^ ^86^ ^]^
LC	Diagnosis	Plasma	UC + Antibody‐modified Au surface	SPR	1 × 10^4^ EVs µL^−1^	8LC + 4NC	^[^ ^122^ ^]^
LC	Diagnosis	Plasma	NA	RCA and CRISPR‐CAS9 based FL	90 × 10^−15^ m	10LC + 10NC	^[^ ^162^ ^]^
LC	Diagnosis	Serum	Antibody‐modified Au surface	AuNPs based SAW sensor	1100 EVs µL^−1^	5LC + 5NC	^[^ ^154^ ^]^
LC	Diagnosis	Urine	Filtration+ Antibody‐modified Au surface	SPR	1 EVs µL^−1^	4LC + 4NC	^[^ ^124^ ^]^
LC	Treatment monitoring	Plasma	Antibody‐modified SiNWs	RNA sequencing	NA	7LC + 9NC	^[^ ^106^ ^]^
LC	Diagnosis	Plasma	CD63‐Aptamers	FP effect	500 EVs µL^−1^	11LC + 6NC	^[^ ^120^ ^]^
LC	Diagnosis and staging	Plasma	SEC	SERS spectrum	NA	43LC + 20NC	^[^ ^137^ ^]^
OVCA	Diagnosis	Plasma	Aptamer‐modified GMR sensor surface	Aptamer‐modified MNPs 2D MoS_2_–Fe_3_O_4_ (MOFE) + GMR sensor	100 EVs	3OVAC + 5NC	^[^ ^151^ ^]^
OVCA	Diagnosis and staging	Plasma	Antibody‐conjugated silica beads in a PDMS chip	Fluorescence labeled antibodies	21 EVs µL^−1^	15OVCA + 5NC	^[^ ^90^ ^]^
OVCA	Diagnosis and staging	Plasma	Antibody‐modified SiO_2_ nanostructure	FL dye (DiO) staining	10 EVs µL^−1^	20OVCA + 20NC	^[^ ^109^ ^]^
PRAD	Diagnosis	Plasma	PS based electrostatic affinity	EpCAM‐aptamer Fe_3_O_4_ NPs based HRP	3580 EVs µL^−1^	3PRAD	^[^ ^95^ ^]^
PRAD	Diagnosis	Serum	Aptamers	GO quenched aptamers	160 EVs µL^−1^	8PRAD + 6NC	^[^ ^93^ ^]^
PDAC	Diagnosis	Plasma	DEP	FL‐labeled Antibodies	NA	20PDAC + 18NC	^[^ ^66^ ^]^
PDAC	Diagnosis	Plasma	Lipid probe‐modified PDMS chip	ddPCR	NA	3PDAC	^[^ ^79^ ^]^
PDAC	Diagnosis	Serum	Antibody affinity	QDs labeled antibodies	1.9 × 10^8^ EVs	12PDAC + 12NC	^[^ ^117^ ^]^
PDAC	Diagnosis	Serum	TiO_2_ microbeads	MS Proteomics	NA	9PDAC + 9NC	^[^ ^87^ ^]^
PDAC	Diagnosis	Serum	PEI based electrostatic affinity	DNA‐conjugated Antibodies + Cy5‐cDNA	NA	7PDAC + 7BRCA + 7NC	^[^ ^163^ ^]^
PDAC	Diagnosis and staging	Serum	Antibody‐modified chip	SERS	0.544 EVs µL^−1^	71PDAC + 32NC	^[^ ^130^ ^]^
SKCM	Treatment monitoring	Plasma	Antibody‐modified PDMS chip	SERS nanotags	NA	15SKCM + 12 NC	^[^ ^132^ ^]^
GC+CRC	Prognosis	Ascites	AuNPs and aptamer affinity	SPR	1500 EVs	12GC + 8CRC	^[^ ^125^ ^]^
LC+PRAD	Treatment monitoring	Plasma	PEG precipitation	Multivalent recognition driving DNA nanomachine	33 EVs µL^−1^	18LC + 8PRAD + 10NC	^[^ ^164^ ^]^
LC+SKCM	Diagnosis	Plasma	Annexin A5‐modified PDMS	NTA (EVs counting)	NA	4LC + 3SKCM + 5NC	^[^ ^83^ ^]^
Cancer^b)^	Treatment monitoring	Plasma	Aptamers thermophoresis	FL‐labeled aptamers	17.6 pg mL^−1^ (PD‐L1)	34Cancer + 22NC	^[^ ^75^ ^]^
PanCancer	Diagnosis and classification	Plasma	NA	Au‐coated TiO_2_ MIO SERS sensor	NA	15PRAD + 15HCC + 15LC + 8CC + 10NC	^[^ ^131^ ^]^
PanCancer	Diagnosis and classification	Serum	Aptamers thermophoresis	FL labeled Aptamers	3300 EVs µL^−1^	60PanCancer + 10NC	^[^ ^73^ ^]^
Brain Injury	Diagnosis	Plasma	Ab‐GluR2‐conjugated 30 nm MNPs	miRNA‐sequencing	NA	30Brain Injury + 30NC	^[^ ^165^ ^]^
Brain Injury	Diagnosis	Plasma	Ab‐CD63/Ab‐A*β*42 capture	SPR	200 EVs	72Brain Injury	^[^ ^166^ ^]^
CVD^c)^	Diagnosis	Plasma	Ab‐CD63‐conjugated microbeads	Au‐based FETs	6.069 × 10^−15^ m (miR‐21)	1 CVD	^[^ ^149^ ^]^

^a)^ Cancer names are abbreviated according to TCGA. BRCA: breast cancer; GBM: glioblastoma multiform; GC: gastric cancer; HCC: hepatocellular carcinoma; HNSCC: head and neck squamous cell carcinoma; LC: lung cancer; OVCA: ovarian cancer; PRAD: prostatic adenocarcinoma; PDAC: pancreatic ductal adenocarcinoma; SKCM: skin cutaneous melanoma; CRC: colorectal cancer

^b)^ This study did not report the cancer type of the patients involved

^c)^ CVD: Cardiovascular diseases.

### 4.4 Single EV Detection

The heterogeneity of EVs is one of the main barriers to analyzing EVs from body fluids. A refined reassessment of EV composition by high‐resolution DGC showed a more limited repertoire of the molecules present in EVs than previously reported.^[^ ^167^ ^]^ Additionally, the *N*‐glycoproteomic and phosphoproteomic data for urine EVs of different sizes suggested that proteins encapsulated in large, medium, and small EVs conceived very distinct glycosylation and phosphorylation levels.^[^ ^168^ ^]^ These results highlight the need for a clear understanding of EV heterogeneity. Ji et al. used a high‐density microchamber to separate every single cell from the others and found that the decrease in CD63+ EVs was associated with the invasive feature of oral squamous cell carcinoma.^[^ ^169^ ^]^ Even though single‐cell EV analysis could provide enough information to characterize EVs originating from different cells, regular population measurements of EVs still masked the heterogeneity of EVs secreted from the same cell. Thus, single EV detection is urgently needed to fully decipher the code of circulating EVs.

**Figure 9:**
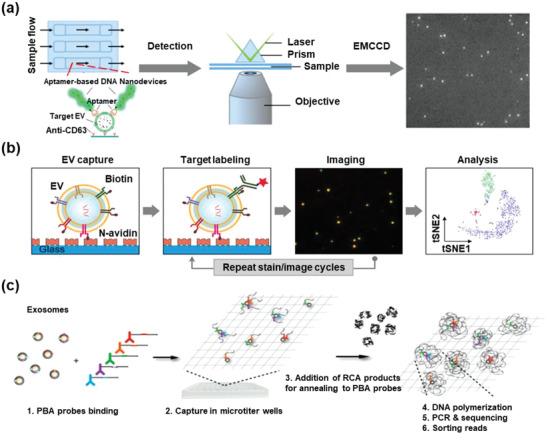
Single EV detection. a) Total internal reflection fluorescence (TIRF) based imaging. Reproduced with permission.^[^ ^170^ ^]^ Copyright 2019, American Chemical Society. b) Multiplex single EV imaging. Reproduced with permission.^[^ ^171^ ^]^ Copyright 2018, American Chemical Society. c) PCR‐based signal amplification of a single EV signal. Reproduced with permission. ^[^ ^174^ ^]^ Copyright 2019, Springer Nature.

Total internal reflection fluorescence (TIRF) microscopy is a sensitive approach suitable for single EV detection. For instance, Chen et al. developed DNA point accumulation for imaging in nanoscale topography (DNA–PAINT) to enable single EV detection.^[^ ^163^ ^]^ Meanwhile, He et al. used an aptamer‐based DNA nanodevice to amplify the fluorescence signal of a single EV to make it detectable by TIRF microscopy (**Figure** **9**a).^[^ ^170^ ^]^ Additionally, Zheng's group developed an ExoELISA chip enabling single EV counting, thereby providing a preliminary solution to single EV detection in a liquid phase‐based manner.^[^ ^77^ ^]^

Multiplex profiling in single‐EV detection is much more challenging. For instance, Lee et al. invented a single EV platform that could capture biotinylated EVs on a neutravidin‐modified surface and detect stationary EVs using fluorescent antibodies. Repeat stain‐image‐quench cycles enable researchers to obtain multiplex profiling of EV surface proteins at a single‐EV level (Figure 9b).^[^ ^171^ ^]^ Using this platform, Fraser et al. provided a single‐EV landscape of circulating EVs in glioblastoma patients to differentiate EVs from different cell origins. They further demonstrated that tumoral EVs are present in <10% of all EVs in the plasma of patients.^[^ ^172^ ^]^ Similarly, another direct single EV imaging system that separates EVs by filtrating, immobilizing, and visualizing EVs via antibodies has also been reported.^[^ ^173^ ^]^

In addition to the stain‐image‐quench‐based platform, Kamali‐Moghaddam's group also developed a single EV detection platform based on next‐generation sequencing (NGS). They dispersed single EVs into microtiter wells and detected them using DNA barcode‐conjugated antibodies, which could be amplified via rolling circle amplification and subsequently detected by NGS and qPCR (Figure 9c).^[^ ^174^ ^]^

Flow cytometry (FCM) is the most commonly used technique for analyzing microscale particles, such as cells, but cannot be used to detect nanoscale EVs directly. To overcome diffraction‐limited FCM by micro‐nanomaterials, many scientists have integrated traditional FCM with microbeads. For example, Melo et al. captured EVs using microsized latex beads and labeled them with fluorescent antibodies to detect GPC1 + EVs from the plasma of patients with cancer.^[^ ^21^ ^]^ Li et al. also developed a microbead‐assisted FCM approach for EV assessment, which enabled rapid detection and molecular phenotyping of patients with cancer.^[^ ^175^ ^]^ In 2018, Tian et al. performed nanoscale FCM (NanoFCM) analysis of EVs, which simultaneously provided protein profiling and sizing data at a single‐EV resolution.^[^ ^176^ ^]^ Various nanoscale microscopic techniques have also been used for EV observation. For instance, atomic force microscopy (AFM) can not only detect a single EV, but also provide nanomechanical information about EVs.^[^ ^177^ ^]^ Additionally, AFM can also be integrated with scanning near‐field optical microscopy (SNOM) to record molecular information simultaneously.^[^ ^178^ ^]^ Nizamudeen et al. used direct stochastic optical reconstruction microscopy (d‐STORM) to characterize a single EV, enabling live imaging of EV uptake in stem cells.^[^ ^179^ ^]^

Generally, single EV detection is regarded as not only the ultimate solution to highly sensitive EV detection, but also a powerful approach to address the heterogeneity of EVs. Even though many technologies have been used to decrease the LoDs of EV detection, obtaining detailed information from a single EV is still a great challenge. Nevertheless, the fine advancement of analytical tools in the future would bring more possibilities for advanced single EV detection technologies.

## 5 Advances in EV Analysis for Liquid Biopsy

In order to use advanced EV detection nanotechnologies for liquid biopsy, deciphering the biomarkers carried on EVs is the last challenge. The detection of EV biomarkers is enabled by coupling specific antibodies or aptamers with advanced sensoring nanotechnologies, as introduced in the last chapter. Different biomarkers carried on EVs can be used in different clinical scenarios. Additionally, to integrate multiple biomarkers into a classification model with the highest efficiency, advanced algorithms have been developed and have facilitated the advancement of EV‐based liquid biopsy.

### 5.1 EV Analysis in Different Clinical Scenarios

Compared to the specimens from traditional biopsy, EVs derived from body fluids could be taken more frequently due to their minimal invasion, which makes them useful across the progress of cancer care, ranging from diagnosis, prognosis, therapy response prediction to treatment monitoring. For the reason that the application of EV detection in cancer diagnosis has been well reviewed,^[^ ^180^, ^181^ ^]^ we mainly focus on the potential applications after diagnosis.

Accurate prognosis prediction of different patients would help clinicians optimize their treatment approaches and design individualized follow‐up and risk‐warning plans. As summarized in **Table** **2**, according to clinically oriented researches with traditional EV detection approaches, most prognostic biomarkers detected in circulating EVs are miRNAs. For lung cancer, Kanaoka et al. revealed the diagnostic and prognostic roles of plasma EV‐derived miR‐451a in a cohort of 285 patients.^[^ ^182^ ^]^ Moreover, EV‐based protein biomarkers such as NY‐ESO‐1, EGFR, and PLAP have also been reported for the prognosis of lung cancer by other clinicians.^[^ ^183^ ^]^ For prostate cancer, plasma EV‐derived miR‐1290, and miR‐375 were identified as prognostic factors.^[^ ^184^ ^]^ Besides, gastrointestinal cancers account for more than half of all cancer cases, and many EV‐based miRNA biomarkers have been identified by different clinical teams. For example, miR‐125b was identified as a prognostic factor for pancreatic and liver cancers.^[^ ^185^, ^186^ ^]^ EV‐derived miR‐19a, miR‐21 were associated with prognosis in colorectal cancer,^[^ ^187^, ^188^ ^]^ and miR‐23b was prognostic in gastric cancer.^[^ ^189^ ^]^ LncRNA is another type of EV‐associated RNA biomarker. ENSG00000258332.1 and LINC00635 were identified as prognostic factors in liver cancer,^[^ ^190^ ^]^ while CRNDE‐h, lncRNA‐GAS5, and HOTTIP were prognostic factors in colorectal cancer and gastric cancer.^[^ ^191^, ^192^, ^193^ ^]^ Other molecules in EVs have also been identified as prognostic factors. Bernard et al. found that KRAS mutations in EV‐derived DNA could be a prognostic factor in pancreatic cancer,^[^ ^194^ ^]^ while Sun et al. found that CPNE3 protein in circulating EVs was prognostic in colorectal cancer.^[^ ^195^ ^]^ Some primary studies on prognosis‐associated EV biomarkers have also been reported by nonclinical researchers. For example, Wu et al. developed an EV‐templated nanoplasmonic technology to detect ascites EVs (Figure 6c) and found that EV‐derived CD14, EpCAM, and MUC1 were all prognostic factors in both gastric cancer and colorectal cancer.^[^ ^125^ ^]^ Generally, most prognostic EV studies were based on RNA detection, which might be due to the maturity and convenience of the qPCR detection technology of RNA molecules. Proteins and DNA biomarkers were also tested, but small molecules, such as lipids, polysaccharides, and various metabolites, were mostly neglected.

**Table 2:** Representative studies of EV analysis throughout the entire treatment process for patients with cancer

Disease	Application	Body fluids	Isolation	Detection targets	Targets category	Patients No.	Ref.
LC	Prognosis	Plasma	UC	miR‐451a	miRNA	285	^[^ ^182^ ^]^
LC	Prognosis	Plasma	Antibody affinity	NY‐ESO‐1, EGFR, PLAP, EpCAM, Alix	Protein	276	^[^ ^183^ ^]^
PRAD	Prognosis	Plasma	PBP	miR‐1290, miR‐375	miRNA	100	^[^ ^184^ ^]^
PDAC	Prognosis	Plasma	PBP	miR‐125b‐5p	miRNA	152	^[^ ^185^ ^]^
PDAC	Prognosis	Plasma	UC	KRAS	DNA mutation	194	^[^ ^197^ ^]^
HCC	Prognosis	Plasma	PBP	miR‐125b	miRNA	128	^[^ ^186^ ^]^
HCC	Prognosis	Serum	PBP	ENSG00000258332.1, LINC00635	lncRNA	60	^[^ ^190^ ^]^
CRC	Prognosis	Plasma	UC	CPNE3	Protein	32	^[^ ^195^ ^]^
CRC	Prognosis	Serum	UC	miR‐19a	miRNA	209	^[^ ^190^ ^]^
CRC	Prognosis	Plasma	UC	miR‐21	miRNA	326	^[^ ^188^ ^]^
CRC	Prognosis	Serum	PBP	CRNDE‐h	lncRNA	148	^[^ ^194^ ^]^
CRC	Prognosis	Plasma	UC	lncRNA‐GAS5	lncRNA	158	^[^ ^192^ ^]^
GC	Prognosis	Plasma	UC	miR‐23b	miRNA	232	^[^ ^189^ ^]^
GC	Prognosis	Serum	NR	HOTTIP	lncRNA	126	^[^ ^193^ ^]^
GC+CRC	Prognosis	Ascites	Aptamer affinity and AuNPs	CD14, EpCAM, MUC1	Protein	20	^[^ ^125^ ^]^
BRCA+LC	Chemotherapy/Radiotherapy Response	Plasma	UC	HSP70	Protein	37	^[^ ^200^ ^]^
ESCC	Chemotherapy/Radiotherapy Response	Saliva	UC	GOLM1‐NAA35	lncRNA	322	^[^ ^201^ ^]^
BRCA	Chemotherapy/Radiotherapy Response	Serum	UC	miR‐21	miRNA	53	^[^ ^202^ ^]^
LC	Targeted Therapy Monitoring (EGFR)	Plasma	PBP	EGFR	RNA	84	^[^ ^199^ ^]^
LC	Targeted Therapy Monitoring (ROS1)	Plasma	Antibody affinity	EpCAM	Protein	7	^[^ ^132^ ^]^
SKCM	Targeted Therapy Monitoring (BRAF)	Plasma	Antibody affinity	MCSP, MCAM, ERBB3, LNGFR	Protein	15	^[^ ^106^ ^]^
LC+PRAD	Immunotherapy Monitoring	Plasma	PBP	PD‐L1	Protein	26	^[^ ^75^ ^]^
Cancer	Immunotherapy Monitoring	Plasma	Aptamers thermophoresis	PD‐L1	Protein	34	^[^ ^164^ ^]^
SKCM	Immunotherapy Monitoring	Plasma	UC	PD‐L1	Protein	44	^[^ ^8^ ^]^

The prediction of treatment response is another application of EV biomarkers. Identification of patients who could benefit from various adjuvant and neoadjuvant therapies would help us to establish a more personalized treatment plan for each patient. Many trials had been conducted to use EV‐based biomarkers in treatment response prediction and monitoring (Table 2). Chemotherapy/radiotherapy is a regular treatment for patients with cancer, but not all patients are sensitive to these approaches. For instance, Chanteloup et al. reported that higher HSP70+ EVs in plasma indicated a worse response to chemotherapy/radiotherapy in both lung cancer and breast cancer.^[^ ^196^ ^]^ Similarly, Rodríguez‐Martínez et al. reported that miRNA‐21, miRNA‐222, and miRNA‐155 from EVs could be used to monitor the treatment response to neoadjuvant chemotherapy.^[^ ^197^ ^]^ Lin et al. demonstrated that salivary EV‐derived chimeric GOLM1‐NAA35 RNA is a potential biomarker for the assessment of therapeutic response in patients with esophageal cancer.^[^ ^198^ ^]^ Compared with cytotoxic treatments such as chemotherapy/radiotherapy, targeted therapy could kill cancer cells with specific molecular characteristics, resulting in a much lower injury to normal cells. However, constant external pressure leads to cloning selection and results in drug resistance. When drug resistance occurs, monitoring the treatment of targeted therapy would help physicians find suitable alternative drugs. Krug et al. found that combining EV‐derived RNA and cfDNA could identify EGFR mutations with 98% sensitivity, largely exceeding the 82% sensitivity of regular cfDNA BEAMing.^[^ ^199^ ^]^ Some nonclinical teams have also tried to use EV detection to monitor the treatment of targeted therapy with a much smaller sample size.^[^ ^106^, ^132^ ^]^ PD‐L1 based immunotherapy is another promising novel treatment that activates immune cells to kill cancer cells. Monitoring the circulating EV‐derived PD‐L1 could reflect the PD‐L1 level of cancer cells, which could be a possible immunotherapy monitoring method to optimize the design and management of treatment plans.^[^ ^8^, ^75^, ^164^ ^]^

Collectively, most EV‐based liquid biopsy studies aim at the diagnosis of cancer, but these technologies could also be applied throughout the entire treatment process for patients with cancer. Many studies have demonstrated that different biomarkers carried on EVs could be used for different clinical scenarios, and much effort has been made in both the prognosis and monitoring of treatment of patients with cancer. Promising results are continuously emerging, but most of those studies were performed with traditional EV isolation and detection approaches, and more collaborations are needed to improve these clinical applications with the help of advanced nanotechnologies.

### 5.2 Machine Learning for EV Analysis

**Figure 10:**
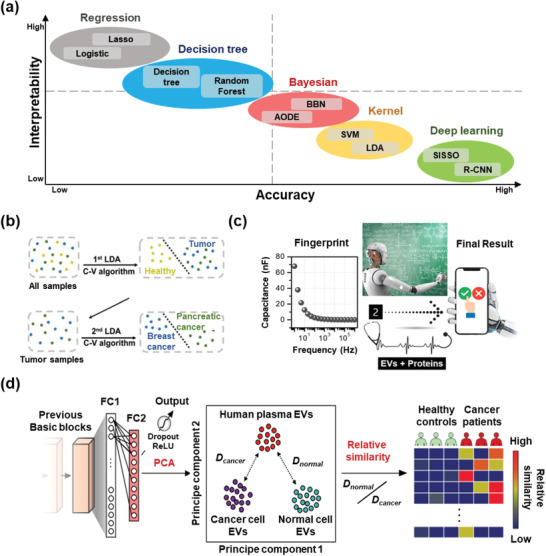
Machine learning for EV analysis. a) The accuracy and interpretability of different algorithms. The accuracy increases and the interpretability decreases along with the increase of complexity. b) Linear discriminant analysis (LDA). Reproduced with permission.^[^ ^163^ ^]^ Copyright 2019, Wiley‐VCH. c) Sure independence screening and sparsifying operator (SISSO). Reproduced with permission.^[^ ^157^ ^]^ Copyright 2020, American Chemical Society. d) Convolutional neural network (CNN). Reproduced with permission.^[^ ^137^ ^]^ Copyright 2020, American Chemical Society.

Generally, it is difficult to achieve satisfactory accuracy by a single biomarker in disease diagnosis, and multiplex profiling of EV‐associated biomarkers is preferred in recent studies. Thus, integrating multivariable data into a classification model with the highest efficiency is crucial. With the rapid development of machine learning technologies, more and more complicated algorithms have been developed, such as linear discriminant analysis (LDA), principal component analysis (PCA), random forests (RF), sure independence screening and sparsifying operator (SISSO), and convolutional neural network (CNN). Recently, various machine learning tools have been used in EV analysis to decipher EV‐based information detected from human body fluids. The accuracy of the algorithms improves along with the increase in complexity, while interpretability decreases correspondingly (**Figure** **10**a). It should be noted that complicated machine learning algorithms would not only bring a black box to the interpretation in modeling but also suffer from the problem of overfitting.

In many EV studies, the use of machine learning algorithms to categorize samples into different groups have been attempted. One of the most popular algorithms in EV studies is LDA, Fisher's linear discriminant method‐based machine learning model, which can be used as a linear classifier by identifying a linear combination of the characteristics of two groups. For example, Liu et al. used a sum signature of seven EV biomarkers to discriminate cancer from noncancerous samples and adopted a two‐step LDA to classify samples into different cancers.^[^ ^73^ ^]^ The LDA voting strategy was also used for DNA–PAINT single EV detection (Figure 10b).^[^ ^163^ ^]^

Other algorithms have also been tested in several EV studies. For instance, Nicoliche et al. tried various methods for modeling EV impedance spectrum data, such as PCA (an algorithm adopts orthogonal transformation to linearly transform the observed values, thus projecting them into a series of linearly unrelated variables), RF (a discriminant method by averaging multiple deep decision trees to reduce variance), and SISSO (an algorithm extracts the most important features describing the properties of the object from immense and correlated feature spaces for regression or classification). All these machine learning models were evaluated, and supervised SISSO was used to successfully discriminate between healthy and diseased samples (Figure 10c).^[^ ^157^ ^]^

CNN is one of the most powerful machine learning algorithms. A CNN model consists of one or more convolution layers and a fully connected layer, as well as an association weight and pooling layer. This structure enables the CNN to make use of the 2D structure of the input data, which significantly improves the utilization efficiency of information and the performance of the model. Shin et al. used a five‐layered residual CNN‐based deep learning model to classify EVs, which outperformed all other traditional algorithms, including PCA, PCA–LDA, partial least‐squares discriminant analysis (PLSDA), and support vector machine (SVM) (Figure 10d).^[^ ^137^ ^]^

In conclusion, machine learning is a very useful toolbox for EV analysis in liquid biopsy. Considering that overfitting problems could possibly be generated, additional cohorts are needed in these studies to further verify those promising models. We believe that the emergence of more powerful machine learning algorithms, such as reinforcement learning, would further improve the performance of EV analysis in liquid biopsy in the near future.

## 6 Conclusion and Perspective

**Figure 11:**
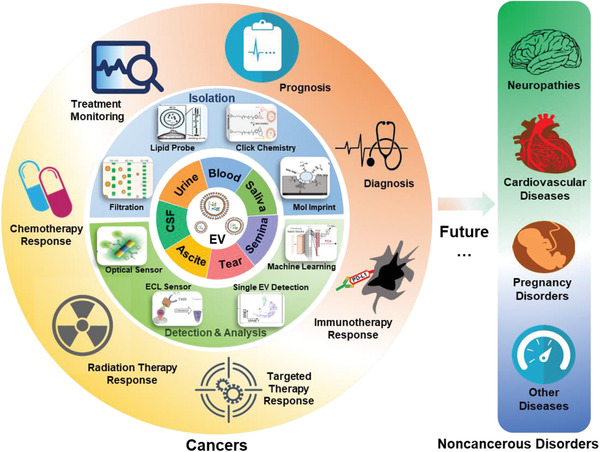
Advanced EV detection nanotechnologies for liquid biopsy. Various nanotechnologies for EV isolation, detection, and analysis have been developed and tested in body fluids, which would be employed for the diagnosis and treatment of different diseases, such as cancers, neuropathies, cardiovascular diseases, and pregnancy disorders.

In this review, we summarize the key advances in recent nanotechnologies for detecting EVs in human body fluids in recent years, as well as the broad applications of EV detection in the diagnosis and treatment of cancers (**Figure** **11**).

For EV isolation, an increasing number of materials scientists prefer to adopt a new strategy by combining structural matching with molecular recognition. This strategy exhibits high efficiency and does not rely on highly sophisticated equipment. The development of microfluidics and nanomaterials in the past decade has accumulated a large number of available tools to improve the topographic interaction between EVs and the capture interface, while basic biochemical research has proposed various molecular recognition patterns between natural EVs and their recipient cells. Recent studies have reported promising performance for EV isolation with ad hoc combinatorial EV capture instruments.^[^ ^103^, ^107^, ^108^ ^]^ However, only a small fraction of these tools have been explored, suggesting that there is still sufficient room for developing highly efficient EV isolation procedures.

For EV analysis, the most impressive progress in the past decade is the application of various biosensors for EV detection. A highly sensitive biosensor is not only important for improving the sensitivity of disease diagnosis, but also offsets the insufficient purity of the isolation procedure. Generally, the development and application of novel signal generators and sensors in EV detection studies have largely reduced the LoDs of EVs, whereas LoDs should be traded off against clinical availability, cost‐effectiveness, and robustness, rather than merely improving LoDs. Moreover, single EV detection approaches not only provide an ultimate LoD, but also propose a good solution to the heterogeneity of EVs. However, research on the molecular composition of single EVs is still in its infancy, which would be boosted along with advances in single‐molecule localization microscopy. The heterogeneity of EVs also leads researchers to prefer multiplex profiling of EV‐associated biomarkers. Various machine learning algorithms provide better performance in EV analysis than a single biomarker. However, the overfitting problem has been largely overlooked in most of these studies. Here, we propose that an additional cohort should be included and tested to verify the machine learning‐based model, if possible.

Detecting EVs and their associated biomarkers in human body fluids is important but challenging, especially when considering the complexity of the physiological and biochemical properties of various body fluids. Most of the newly developed EV detection technologies were only tested in plasma or serum (Table 1), and some researchers even used mimetic serum samples to evaluate the LoDs. However, the reported LoDs can be distorted in liquids with different compositions. For instance, Lyu et al. demonstrated that fluorescent LoD dramatically increased when PBS was changed to plasma.^[^ ^121^ ^]^ Similarly, the aptamer‐based EV detection reported by Yu et al. exhibited a 100 times larger LoD in serum than that in PBS.^[^ ^203^ ^]^ Thus, although the LoDs of different EV detection tools summarized in Table 1 seem promising, we still need to be cautious that these LoDs were not evaluated under the same conditions. Here, we suggest that along with the pursuit of better LoDs, the robustness and anti‐interference performance should be considered, and a detection approach compatible with all body fluid environments would be the game‐changer for EV‐based liquid biopsy.

In addition to the diagnosis of various cancers, EV‐based liquid biopsy would also be a powerful approach for detecting noncancerous diseases. The diversity of body fluids inspires clinicians to investigate the role of EVs in the diagnosis of diseases closely related to specific body fluids (e.g., neuropathy and CSF, pregnancy disorders and amniotic fluid, nephropathy and urine). Hence, some pilot studies have highlighted the possible application of EV‐based technologies in the diagnosis of noncancerous diseases. For example, miR‐939, C1QA, C5, APOC3, CST3, SERPINF2, and CD14 derived from circulating EVs were increased in patients with myocardial ischemia/infarction,^[^ ^201^, ^204^, ^205^ ^]^ and CST3, CD14, miR‐9, and miR‐124 were indicators of ischemic stroke.^[^ ^201^, ^206^ ^]^ Additionally, miR‐193b, miR‐1, miR‐19b‐3p, etc. were found specifically elevated in the CSF of patients with Alzheimer's disease and Parkinson's disease.^[^ ^207^, ^208^ ^]^ Moreover, Eissa et al. and Yu et al. found that urine EV‐derived miR‐133b, miR‐342, miR‐30, and miR‐200b were abnormally elevated in patients with diabetic nephropathy and renal fibrosis, respectively.^[^ ^209^, ^210^ ^]^ It is worth noting that in most studies based on EV detection in the diagnosis of noncancerous diseases, the focus was EV miRNAs and EV proteins. Other possible biomarkers such as DNA, mRNA, lncRNA, and lipids need to be investigated in the future.

It is worth noting that most EV biomarkers in liquid biopsy were detected in plasma/serum. Compared with other body fluids, plasma exhibits the advantages of universal availability and high stability owing to the homeostatic regulatory system of blood. According to their various physical and chemical characteristics, standardization of sample pre‐processing is a great challenge in nonplasma body fluid‐based EV detection. For sample pre‐processing, high dilution before centrifugation or filtration is a critical step to overcome the viscosity, and the optimized dilution ratio of different body fluids should be explored in bile, saliva, and cerebrospinal fluid. Microbial contamination is also a problem that should be carefully removed in the sample pre‐processing procedures, especially when handling ascites fluid, urine, bile, prostatic fluid, and semina. Proper sampling timing could also avoid the problem of heterogeneity in EV detection based on urine or saliva.

For the reference materials in EV detection, the regular approach is to add exogenous EV‐mimic nanoparticles or macromolecules to the fluid to be tested. Recently, the selection of reference materials mainly depends on downstream detection methods rather than on the type of body fluids. For the detection of special EV biomarkers, such as EV miRNAs, exogenous nonhuman miRNAs (e.g., cel‐39‐3p) were used as reference materials for the calibration of EV miRNA quantification.^[^ ^10^ ^]^ For EV counting and size measurement, polystyrene or silica nanospheres are widely used in NTA and nanoFCM assays.^[^ ^211^ ^]^ However, using these artificial materials as a reference would introduce inevitable bias because they are only similar in size, but quite different in optical, mechanical, and chemical properties as compared to natural EVs. Liposomes exhibit physical and chemical properties that are similar to those of EVs and thus can act as a better choice of EV reference materials than other solid nanospheres. Considering that the manufacture of liposomes of different sizes and compositions has matured over the past decade, we believe that the application of optimized liposomes as reference materials in EV detection would improve the comparability and repeatability of this field. Industrial scientists have proposed a higher requirement for EV reference materials. Jonathan et al. suggested that the serial ultracentrifugation step incorporation of a density gradient obtained high‐purity EVs, which would be an ideal reference material for both EV manufacturing and detection.^[^ ^212^ ^]^

In conclusion, advanced nanotechnologies and analytical methodologies, such as machine learning, will provide abundant possibilities for the development of clinically applicable EV detection approaches. We believe that the close interdisciplinary cooperation between materials scientists and clinicians in EV‐based liquid biopsy would greatly optimize existing diagnosis and treatment processes in the future.

## Conflict of Interest

The authors declare no conflict of interest.
